# Lack of Adipocytes Alters Hematopoiesis in Lipodystrophic Mice

**DOI:** 10.3389/fimmu.2018.02573

**Published:** 2018-11-13

**Authors:** Anne Wilson, He Fu, Mariano Schiffrin, Carine Winkler, Meriem Koufany, Jean-Yves Jouzeau, Nicolas Bonnet, Federica Gilardi, François Renevey, Sanjiv A. Luther, David Moulin, Béatrice Desvergne

**Affiliations:** ^1^Department of Oncology, University of Lausanne, Epalinges, Switzerland; ^2^Faculty of Biology and Medicine, Center for Integrative Genomics, Genopode, University of Lausanne, Lausanne, Switzerland; ^3^IMoPA, UMR7365 CNRS-Université de Lorraine, Vandœuvre-lès-Nancy, France; ^4^Division of Bone Diseases, Department of Internal Medicine Specialties, Faculty of Medicine, Geneva University Hospital, Geneva, Switzerland; ^5^Department of Biochemistry, Center for Immunity and Infection, University of Lausanne, Epalinges, Switzerland; ^6^CHRU de Nancy, Contrat d'interface, Vandœuvre-lès-Nancy, France

**Keywords:** lipodystrophy, PPARγ null mice, AZIPtg/+ mice, bone marrow adipocytes, hematopoiesis, extramedullary hematopoiesis, inflammation, non-cell autonomous alteration of hematopoiesis in PPARγ null mice

## Abstract

Adult hematopoiesis takes place in the perivascular zone of the bone cavity, where endothelial cells, mesenchymal stromal/stem cells and their derivatives such as osteoblasts are key components of bone marrow (BM) niches. Defining the contribution of BM adipocytes to the hematopoietic stem cell niche remains controversial. While an excess of medullar adiposity is generally considered deleterious for hematopoiesis, an active role for adipocytes in shaping the niche has also been proposed. We thus investigated the consequences of total adipocyte deletion, including in the BM niche, on adult hematopoiesis using mice carrying a constitutive deletion of the gene coding for the nuclear receptor peroxisome proliferator-activated receptor-γ (PPARγ). We show that *Pparg*^Δ/Δ^ lipodystrophic mice exhibit severe extramedullary hematopoiesis (EMH), which we found to be non-cell autonomous, as it is reproduced when wild-type donor BM cells are transferred into *Pparg*^Δ/Δ^ recipients. This phenotype is not due to a specific alteration linked to *Pparg* deletion, such as chronic inflammation, since it is also found in AZIP^tg/+^ mice, another lipodystrophic mouse model with normal PPARγ expression, that display only very moderate levels of inflammation. In both models, the lack of adipocytes alters subpopulations of both myeloid and lymphoid cells. The CXCL12/CXCR4 axis in the BM is also dysregulated in an adipocyte deprived environment supporting the hypothesis that adipocytes are required for normal hematopoietic stem cell mobilization or retention. Altogether, these data suggest an important role for adipocytes, and possibly for the molecular interactions they provide within the BM, in maintaining the appropriate microenvironment for hematopoietic homeostasis.

## Introduction

Bones and hematopoiesis are intimately linked. Adult hematopoiesis takes place in the bone cavity, where a variety of cells and molecular contacts create a niche allowing hematopoietic stem cells (HSCs) to undergo cell division and differentiation in a highly regulated manner. The concept of a stem cell niche was first coined by Schofield, who hypothesized that the cellular environment in the bone compartment creates multiple cell-cell contacts that are crucial for HSC function (1). Depending on the location in the bone cavity, the different cell types involved and different functions proposed, BM niches are described as endosteal, reticular, sinusoidal or perivascular, mainly involving osteoclast precursors, osteoblast and spindle-shaped osteoblast precursors (SNO), CXCL12-abundant-reticular (CAR) cells, Nestin+ mesenchymal stromal cells (MSCs), E-selectin+ endothelial cells, LeptinR+ perivascular stromal cells, and non-myelinating Schwann cells, respectively [reviewed in (2–4)].

The role of adipocytes, present in large numbers in the BM cavity, remains disputed. Adipocytes, which are the specialized cells of adipose tissue, store energy in the form of lipids, and release it when required by the organism. Adipocytes also secrete cytokines known as adipokines that participate in endocrine-mediated homeostasis (5). Both gain of adipose tissue, as in obesity, and the generalized lack of adipose tissue (generalized lipodystrophy), such as is seen in Berardinelli-Seip syndrome, causes metabolic disorders such as hypertriglyceridemia, metabolic syndrome, and type 2 diabetes (6, 7). While most adipocytes are found within depots forming the diverse adipose tissues, some of them are also found in substantial numbers in a less organized manner, particularly within the BM where their (local) role is less well characterized (8). The first link between adipocytes and the bone microenvironment is the fact that both adipocytes and osteoblasts are derived from a common mesenchymal progenitor, and their respective production is due to a balance between adipogenesis and osteoblastogenesis. A more direct contribution of adipocytes to the stem cell niche in the BM has been previously explored, albeit with contradictory results. First, using leptin deficient mice (*ob/ob* mice), Claycombe et al. showed that supplementation with leptin, a major adipokine secreted by adipocytes, rescued appropriate levels of lymphopoiesis and myelopoiesis in the BM (9). Second, a combination of *in vitro* and *in vivo* experiments has suggested that adiponectin, another adipokine expressed by adipocytes in the BM, is required for optimal HSC growth (10, 11). Third, BM adipocytes also secrete Stem Cell Factor, which contributes to restoring hematopoiesis after irradiation in the long bones but not in the vertebral bones (12). Finally, experiments performed in AZIP-F1 (AZIP^tg/+^) transgenic mice carrying a C/EBP dominant negative transgene that induces deletion of mature adipocytes, showed improved marrow engraftment after irradiation, suggesting that in this specific context adipocytes are negative regulators of hematopoiesis (10, 13). A similar negative effect is also proposed when adipocytes overfill the medullary space upon BM failure in Fanconi Anemia (14).

In the present report, we reveal a novel aspect of the cross-talk between hematopoiesis and adipocytes, by exploiting a generalized lipodystrophic mouse model carrying a constitutive total-body deletion of the nuclear receptor peroxisome proliferator-activated receptor-γ (PPARγ) (15, 16). *Pparg*^Δ/Δ^ mice show a complex phenotype including total lipoatrophy, increased lean mass, and hypermetabolism. They develop severe type 2 diabetes, characterized by hyperglycemia, hyperinsulinemia, polyuria, and polydipsia (personal communication, manuscript in preparation). Herein, we demonstrate that the total lack of adipocytes is accompanied by extramedullary hematopoiesis (EMH), which is defined as the production of blood cells occurring outside of the BM, mainly in the liver and spleen (17). We further evaluate the causes of this EMH and provide new insights in the role of adipocyte signaling in hematopoiesis.

## Materials and methods

### Mice

Genotype designations in this work follow the rules recommended by the Mouse Genome Database Nomenclature Committee. Procedures using mice were authorized by the Cantonal Commission for Animal Experimentation of the Canton of Vaud and carried out in accordance with the International Guiding Principles for Biomedical Research Involving Animals. *Sox2-Cre* transgenic mice (*Sox2-Cre* ^tg/+^; Tg(Sox2-cre)1Amc/J), CD45.1+ (B6.SJL-*Ptprc*^*a*^*Pepc*^*b*^/BoyJ) mice (Jackson Laboratory, Bar Harbour, MA), and *ob/ob* mice were kept in the University of Lausanne Animal Facility. Construction of the *Pparg* floxed (hereafter referred to as *Pparg*^*fl*^) and *Pparg*-null alleles resulting from Cre recombination (hereafter referred to as *Pparg*^Δ^), as well as the mating strategy for the generation of *Sox2-Cre*^tg/+^Pparg^*emΔ*/Δ^ (*Pparg*^Δ/Δ^) mice and their control littermates (CTL) with no *Sox2-Cre* transgene but two functional *Pparg* alleles (*Pparg*^*fl*/+^) have been previously described (16, 18). This strategy ended up with a conditional epiblast-specific deletion of *Pparg* mediated by the *Sox2-Cre* transgene. The preservation of *Pparg* expression in the trophoblast (16) circumvented the embryonic-lethality of homozygous PPARγ knockout mice due to a placental defect (15, 16). Normal placental development allows *Sox2-Cre*^tg/+^*Pparg*^emΔ/Δ^ pups to be born, and as expected, these mice are totally deprived of any form of adipose tissue. Both male and female mice 12–22 weeks of age were used. AZIP/F1 mice on an FVB background [Tg(AZIP/F)1Vsn; hereafter referred to as AZIP^tg/+^] and corresponding wild-type FVB controls were obtained from Charles Vinson and the colony was raised as previously described (19).

No *Pparg* expression could be detected in the long bones of *Pparg*^Δ/Δ^ mice and the total lack of adipocytes in the BM in these two models, was confirmed by using Resistin, an adipokine specifically expressed by adipocytes and, herein, used as a surrogate marker for the presence of mature adipocytes. Expression of Resistin was indeed barely measurable above the detection threshold in mRNA extracted from *Pparg*^Δ/Δ^ bones, and at very low levels in AZIP^tg/+^ bones (Supplementary Figure S1).

### Flow cytometry

BM cell suspensions from all mouse strains described above were prepared by crushing the long bones (2 femurs and 2 tibias per mouse) into DMEM/3% FCS. Bone fragments were removed by filtration through 40-μm filters. Splenocyte suspensions were obtained by mashing the organs through sieves into DMEM/3% FCS, washing by centrifugation and filtering through a 40-μm filter cap. Liver hematopoietic mononuclear cells were obtained by mashing entire livers through sieves, after which the cells were washed in DMEM/3% FCS and centrifuged using a Percoll (GE Healthcare) gradient (40% Percoll layered over 80% Percoll) for 30 mins at 2,000 rpm to remove hepatocytes and other non-hematopoietic cells. Cells localized at the interface were recovered, diluted in DMEM/3% FCS, centrifuged and filtered through 40-μm filter caps. Single-cell suspensions were stained as previously described (20). Monoclonal antibody conjugates used for flow cytometry are listed in Supplementary Table S1. Cells were analyzed on a 5-laser LSR II cytometer equipped with 355, 405, 488, 561, and 640-nm lasers (Becton Dickinson, San Jose, CA**)**, and the data were analyzed with FlowJo V9 software (TreeStar, Ashland, OR).

### Colony forming cell (CFC) assay

Splenocyte cell suspensions were obtained by mashing the spleen through sieves into DMEM/3% FCS, washing by centrifugation then filtering through 70-μm filter caps. BM cells suspensions were obtained as described above. Splenocytes (3 × 10^5^) and BM cells (2 × 10^4^) were seeded into 35 mm dishes in Mouse Methylcellulose Complete Media containing cytokines/growth factors such as EPO, IL-3, IL-6, SCF, or the combination thereof according to the manufacturer's instructions (R&D Systems). After 10–12 days of culture at 37°C in 5% CO_2_, Colonies were identified by eye with phase-contrast microscopy.

### BM transplantation

BM chimeras were prepared as previously described (21). Briefly, 12-week old host mice (CD45.1+) were lethally irradiated (1000 rads) and reconstituted with 10^7^ T-depleted BM cells (CD45.2+) from either *Pparg*^Δ/Δ^ mice or their littermate controls (CTL). For reverse chimeras, 12-week old host mice (*Pparg*^Δ/Δ^ or CTL) were reconstituted with 10^7^ T-depleted BM cells from CD45.1+ wild-type controls. Hematopoietic reconstitution was assessed by FACS staining of ficoll-purified peripheral blood cells 6 weeks after transfer as described above. Mice were euthanized and analyzed 12 weeks after transfer. Owing to variable reconstitution efficiency between animals, the BM chimera results were expressed as the percentage of donor-derived cells.

### Quantitative RT-PCR

Total RNA was isolated from long bones (bone fragments and BM combined), liver, and spleen, using TRIzol LS reagent (ThermoFisher, Waltham, MA) and purified with the RNeasy kit (Qiagen, Hilden, Germany). RNA quality was verified by chip electrophoresis (Agilent 2100 Bioanalyzer; Santa Clara, CA), and the concentration was determined using Nanodrop (Wilmington, DE). Total RNA (500 ng to 1 μg) was reverse-transcribed using the iScript™ cDNA Synthesis Kit (Bio-Rad Laboratories, Hercules, CA) according to the manufacturer's instructions. Real-time PCR was performed with SYBR® Green PCR mastermix using an ABI PRISM® 7900 PCR machine (ThermoFisher). The results were normalized to the levels of Actin beta (*Actb*). For primer sequences, see Supplementary Table S2.

### Histology

Spleen were fixed in 4% paraformaldehyde and paraffin embedded. Four micro meter paraffin sections were stained with hematoxylin and spleen area as well as white pulp (WP) area were calculated by measuring spleen or WP surface area, using ImageJ software (http://rsbweb.nih.gov/ij/). Red pulp (RP) area was calculated as total spleen area minus WP areas. One representative section per spleen and 3 spleens were analyzed per genotype.

Immunohistochemistry was performed on 8 μm-thick frozen sections of OCT-embedded spleen, which were fixed using acetone followed by primary and secondary antibodies or streptavidin (found in Supplementary Table 1), as described previously (22). Images were acquired with a Zeiss Axioplan microscope and treated with Photoshop (Adobe) or Image J opensource software.

### Serum levels of parathyroid hormone (PTH) and inflammatory markers

Blood was obtained by cardiac puncture immediately after euthanasia. After clotting and centrifugation, serum was collected and stored at −80°C until use. Parathyroid hormone (PTH) was measured by ELISA according to the manufacturer's protocol (Stoughton, MA; LifeSpan Biosciences, Inc., Seattle,WA). Serum levels of Serum Amyloid A (SAA), IL-1β and IL-6 were measured using commercial ELISA kits (Bio-techne, Abingdon, UK). Assays were run in duplicate using adequate dilution buffer (for SAA, sera were diluted between 1:200 and 1:2,000; for IL-1β and IL-6, sera were used pure or diluted 1:2), according to the manufacturer's protocol. A four-parameter logistic formula was used to calculate the sample concentrations from the standard curves. Limit of quantification was 0.022 ng/ml for SAA (manufacturer's data).

### Statistical analyses

The statistical analysis was performed using GraphPad Prism 6 software. Two-group comparisons were performed using Student's *t*-tests or Mann-Withney *U*-test for non-parametric data, as indicated. All data are presented as mean ± SEM.

## Results

### Significant increase in hematopoietic cells in the spleen and liver of lipodystrophic *Pparg*^Δ/Δ^ mice

**Figure 1:**
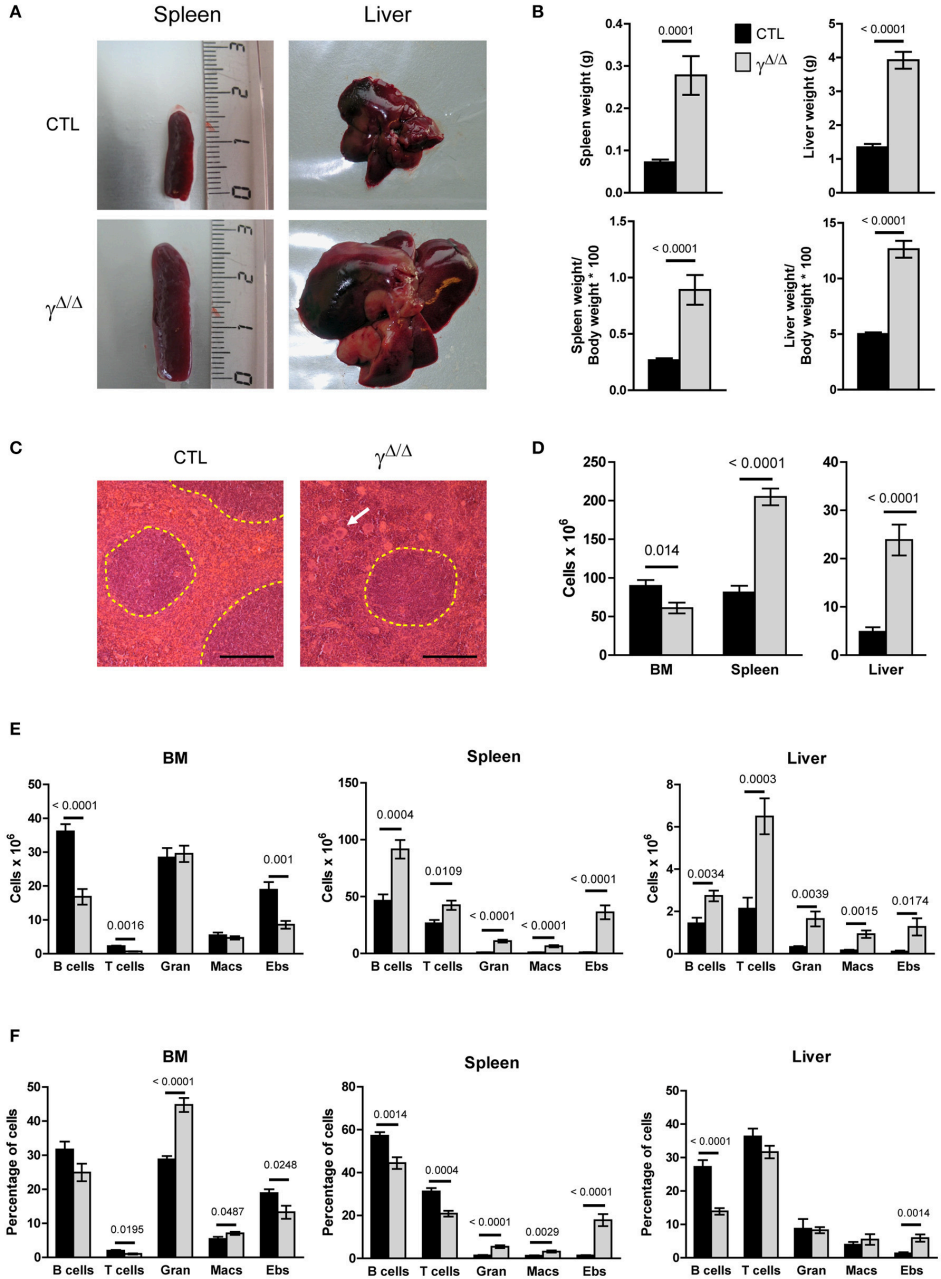
Evaluation of hematopoiesis-derived cell populations in the bone marrow (BM) and peripheral organs of Pparg^Δ/Δ^ mice. **(A)** Representative photographs of the spleens and livers of *Pparg*^Δ/Δ^ (γ^Δ/Δ^) mice (lower panels) and littermate control (CTL) mice (upper panels). **(B)** Spleen and liver weight in grams (top panels) and expressed as % of the body weight (bottom panels). **(C)** Hematoxylin & eosin staining of spleen sections; White pulp areas are circled by a dotted line. The white arrow indicates one of the numerous megakaryocytes present in the red pulp in *Pparg*^Δ/Δ^ mice. The black bar indicates 200 micrometers. **(D)** Total hematopoietic cell numbers in the BM (2 tibias and 2 femurs from each mouse), spleen, and liver of control and *Pparg*^Δ/Δ^ mice. Mean ± SEM, *n* = 7–8 mice per genotype. **(E)** Total numbers of mature hematopoietic cell subsets in the BM (left panel), spleen (middle panel) and liver (right panel) of control (dark bars) and *Pparg*^Δ/Δ^ (light bars) mice. **(F)** Same as in **(E)**, with cell numbers expressed as a % of the total cell number in the corresponding organ. T cells (CD3^+^); B cells (B220^+^); Gran: granulocytes (Gr1^+^CD11b^+^), Macs: macrophages (Gr1^−^CD11b^+^), Ebs: erythroblasts (Ter119^+^CD71^+^); Mean ± SEM *n* = 7–8 mice per genotype. All significant *p*-values are indicated above the corresponding bars.

To explore both the systemic and local involvement of adipose tissue, we characterized the hematopoietic phenotype of mice carrying a constitutive deletion of the two *Pparg* alleles. We have previously shown that the ablation of PPARγ expression leads to the total absence of both white and brown adipose tissue (18) and the development of various metabolic disorders, which include the early onset of a type 2 diabetes [(23) and unpublished observations]. Adult *Pparg*^Δ/Δ^ mice had significantly enlarged spleens and livers, both in volume and weight (Figures 1A,B). Histological analyses revealed an alteration of the red pulp of the spleen, with an increase in the red pulp compartment size (Supplementary Figures S2A,B) and the presence of numerous and large polynuclear cells corresponding to megakaryocytes (Figure 1C). The total surface occupied by the white pulp was similar in the spleen sections from control and *Pparg*^Δ/Δ^ mice while the average white pulp cords were smaller (Supplementary Figures S2A,B). However, the overall perturbation might lead to an underestimation of the white pulp. Immunohistochemical characterization of the spleen further showed that the segregation of various immune cells into the red and white pulp compartment as well as into the B and T zone of the white pulp was not significantly altered in spleens of *Pparg*^Δ/Δ^ mice. Similarly, only a mild reduction in splenic germinal centers was observed (Supplementary Figures S2C–E). During fetal life the liver and spleen are the major sites of hematopoiesis, and the perturbations observed, particularly the presence of numerous megakaryocytes, are suggestive of altered hematopoiesis.

Consistent with a perturbation of hematopoiesis, significant increases in total hematopoietic mononuclear cell numbers were observed in the liver and spleen of Pparg^Δ/Δ^ mice (Figure 1D). Flow cytometric analysis of the major hematopoietic subsets in these organs showed significant increases (10-fold or more) in the numbers of granulocytes, macrophages and erythroblasts.

In contrast to these peripheral organs, the total numbers of hematopoietic cells in the BM of Pparg^Δ/Δ^ mice were marginally decreased compared to the controls (Figure 1D). While the numbers of granulocytes and macrophages in the BM did not differ in Pparg^Δ/Δ^ mice compared to the controls, lymphopoiesis was affected with numbers of B cells and T cells reduced 1.5-fold in Pparg^Δ/Δ^ mice, and erythroblasts decreased ~two-fold (Figures 1E,F). However, this hematopoietic cell dysregulation was associated with only minor changes in the peripheral blood counts (Supplementary Figure S3). Thus, while BM hematopoiesis is mildly altered in the absence of PPARγ, substantial increases in numbers of myeloid (granulocytes and macrophages) and erythroid (erythroblasts and RBCs) lineage cells are observed in peripheral hematopoietic organs such as the liver and spleen.

### Lipodystrophic *Pparg*^Δ/Δ^ mice exhibit active extra-medullary hematopoiesis

To determine whether the massive increase in hematopoietic cells observed in the peripheral organs was due to a local increase in their production, we assessed the number of HSCs and progenitor cells in these organs as well as in the BM. Under homeostatic conditions, adult hematopoiesis occurs almost exclusively in the BM, where mature hematopoietic lineages are normally produced from HSCs and progenitor cells (Supplementary Figure S4) located in specialized BM niches (24). However, under certain conditions (such as BM failure, myelostimulation, or inflammation), substantial numbers of HSCs and multi-potent progenitor cells (MPPs), as well as developing myeloid and erythroid lineages, can be found in peripheral organs such as the spleen and liver, contributing to an extramedullary hematopoiesis (EMH) [reviewed in (17)].

**Figure 2:**
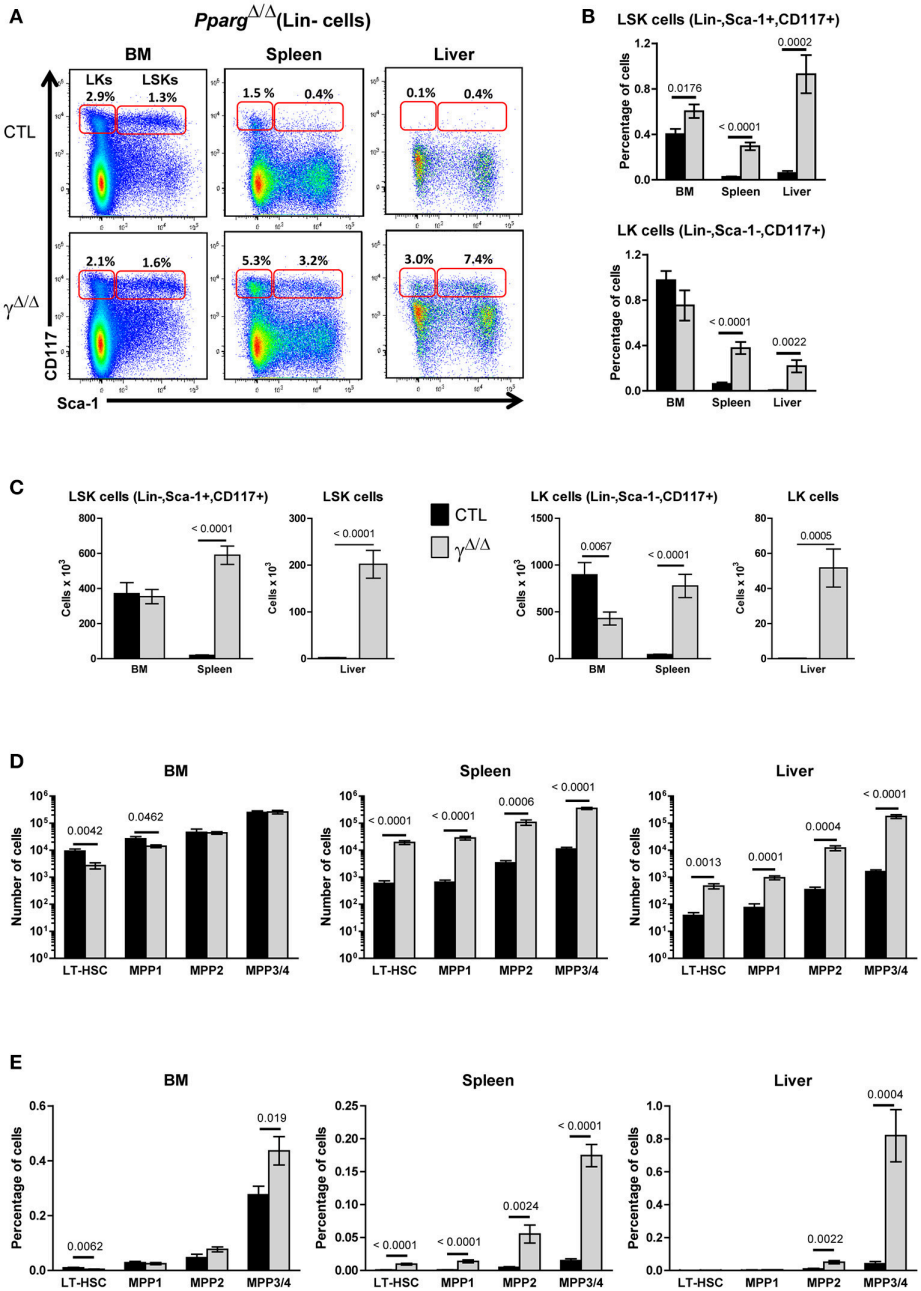
Hematopoietic cell populations in the bone marrow (BM) and peripheral organs of Pparg^Δ/Δ^ mice. **(A)** Representative FACS plots showing Sca-1 vs. CD117 staining on lineage-negative BM (left panels), spleen (middle panels), and liver (right panels) cells from littermate control (CTL, upper panels) or *Pparg*^Δ/Δ^ (lower panels) mice. The red frames on the left and right of each plot indicate the gating and numbers of LK (Lineage-negative, Sca-1^−^CD117^+^) and LSK (Lineage-negative, Sca-1^+^CD117^+^) cells, respectively, as a percentage of lineage-negative cells from each organ. **(B)** Histograms showing the percentage of LSK (top panel) and LK (bottom panel) cells, with respect to the total cell numbers in the BM, spleen and liver of control (dark bars) and *Pparg*^Δ/Δ^ (light bars) mice. Mean ± SEM, *n* = 7–8 mice per genotype. **(C)** Same as in **(B)**, expressed as absolute numbers of LSK (left panels) and LK cells (right panels). **(D)** Quantification of LT-HSC (CD34^−^CD150^+^CD48^−^), MPP1 (CD34^+^CD150^+^CD48^−^), MPP2 (CD34^+^CD150^+^CD48^+^), and MPP3/4 (CD34^+^CD150^−^CD48^+^) subsets in the LSK population of the BM, spleen, and liver from control (dark bars) and *Pparg*^Δ/Δ^ (light bars) mice. Mean ± SEM, *n* = 7–8 mice per genotype. **(E)** Same as in **(D)**, expressed as a percentage of the total cell number in the corresponding organ. All significant *p*-values are indicated above the corresponding bars.

To evaluate and identify cells belonging to the different hematopoietic precursor populations, we analyzed by flow cytometry the LSK (Lin^−^, Sca-1^+^, cKit-r^+^) and LK (Lin^−^, Sca-1^−^, cKit-r^+^) populations. The LSK subset contains long-term (LT-) and short-term (ST-) HSCs, and several Multi-Potent Progenitors (MPPs) populations, while the LK subset contains the Common Myeloid Progenitors (CMPs), and gives rise to both Megakaryocyte Erythroid Progenitors (MEPs) and Granulocyte Monocyte Progenitors (GMPs; see also Supplementary Figure S4). Significant increases in both the relative proportion and total numbers of LSK and LK cells were observed in the spleen and liver of *Pparg*^Δ/Δ^ mice compared to control mice. In contrast, the absolute and relative number of LSK and LK cells in the BM of *Pparg*^Δ/Δ^ mice were modestly affected (Figures 2A–C).

To further identify subsets present in the LSK population, we analyzed the distribution of the markers CD34, CD48, and CD150, which define LT- and ST-HSCs as previously described [(20); Supplementary Figure S4). Although LT-HSC (CD34^−^ CD48^−^ CD150^+^) and MPP1 (ST-HSC; CD34^+^ CD48^+^ CD150^+^) subsets were marginally decreased in the BM, the MPP2 (CD34^+^ CD48^−^ CD150^+^) and MPP3/MPP4 (CD34^+^ CD48^+^ CD150^−^ CD135^+/−^) subsets were unchanged (Figure 2C). Thus, the HSC and MPP subsets were largely unaffected in the BM of mice lacking PPARγ. In contrast, a global 10- to 100-fold increase in LT-HSC and all MPP subsets (MPP1-4) was observed in the spleen and liver of *Pparg*^Δ/Δ^ mice with no changes in their relative proportions compared to those seen in the BM of wild-type controls. These important increases were still observed when calculated as a % of the total cell number in these two organs (Figures 2D,E). The clonogenic potential of these hematopoietic precursor populations found in the spleen was also confirmed by performing a Colony Forming Unit assay on BM and spleen cells (Supplementary Table S3).

As B cells and T cells are derived from hematopoietic progenitors via a Common Lymphoid Precursor (CLP), which is defined as Lin^−^, Sca-1^lo^, cKit-r^lo^ and CD27^+^, CD127^+^, CD135^+^(25), we quantified the proportion and number of CLPs in the BM and spleen of control and *Pparg*^Δ/Δ^ mice. No significant changes were observed in the proportion or in the number of CLPs in the BM and spleen of Pparg^Δ/Δ^ compared to control mice (data not shown).

Taken together, the increases observed in these HSC and progenitor cell subsets along with the increase in mature myeloid and erythroid subsets in peripheral organs are consistent with a significant EMH occurring in *Pparg*^Δ/Δ^ mice.

### The EMH observed in *Pparg*^Δ/Δ^ mice is non-cell autonomous

**Figure 3:**
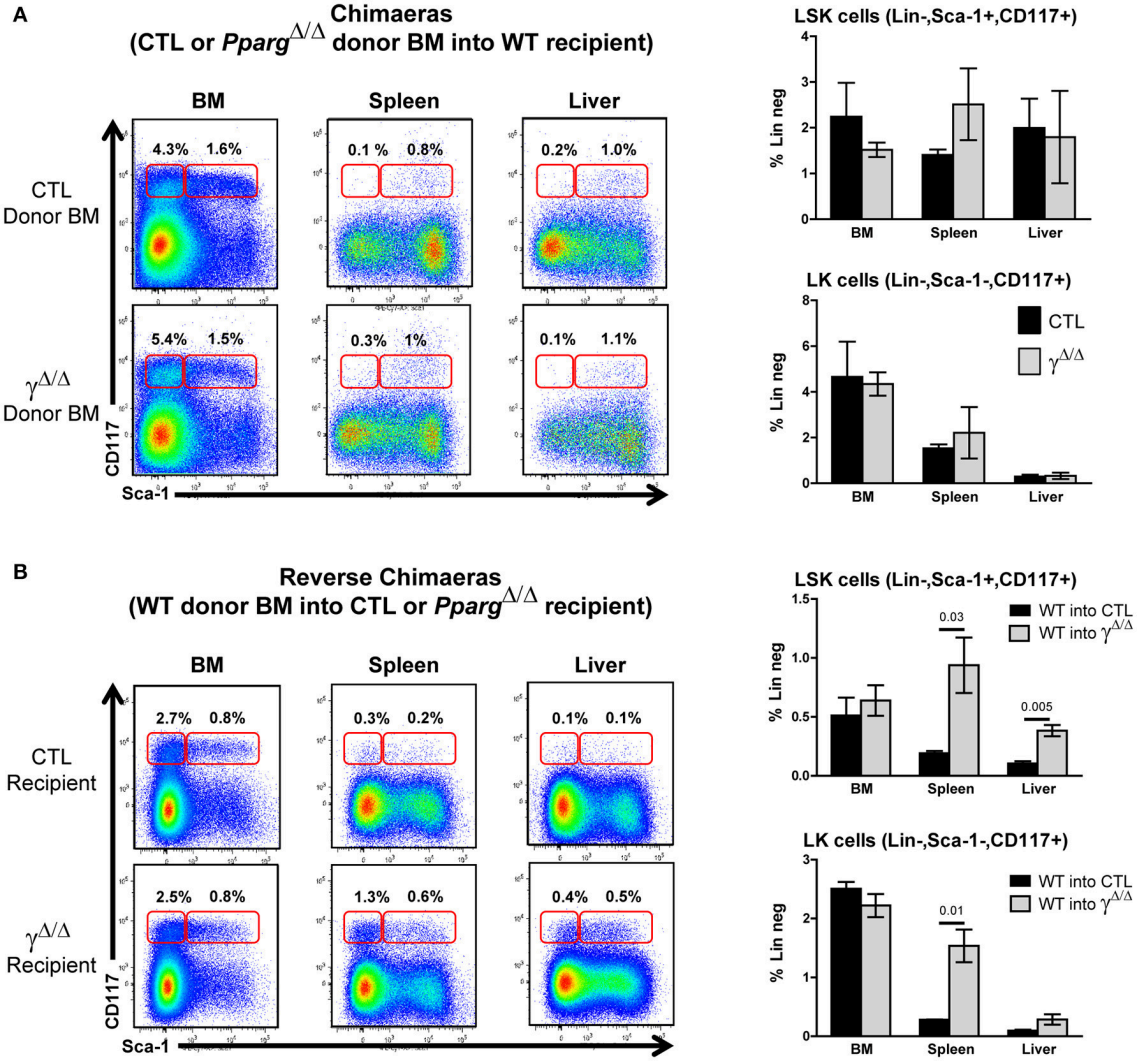
Extramedullary hematopoiesis is non-cell autonomous. **(A)** Chimeras using wild-type recipient: Control or *Pparg*^Δ/Δ^ donor BM (both CD45.2^+^) was transferred into wild-type (WT) recipient CD45.1^+^ mice. Hematopoietic reconstitution was evaluated by FACS analysis 3 months after lethal γ-radiation and i.v. transfer of T-depleted BM. Left panels: representative FACS plots of Sca-1 vs. CD117 (cKit-r) on lineage-negative donor (CD45.2^+^) BM (left), spleen (middle) or liver (right) cells from mice reconstituted with control BM (top row) or *Pparg*^Δ/Δ^ BM (lower row). The red frames on the left and right of each plot indicate the gating and percentage of LK (Lineage-negative, Sca-1^−^CD117^+^) and LSK (Lineage-negative, Sca-1^+^CD117^+^) cells, respectively. Right panels: Relative proportions (in %) of LSK cells (top panel) and LK cells (bottom panel) over the total cell population of the BM, spleen, and liver. Dark bars represent donor control BM, and light bars represent donor *Pparg*^Δ/Δ^ BM, both transplanted into WT host mice. Mean ± SEM, *n* = 3 mice per genotype. There are no significant *p*-values. **(B)** Reverse chimeras: Wild-type (WT) control donor BM (CD45.1^+^) was transferred into CTL or *Pparg*^Δ/Δ^ recipient CD45.2^+^ mice. Left panels: representative FACS plots of Sca-1 vs. CD117 (cKit-r) on lineage-negative donor (CD45.1^+^) BM (left), spleen (middle), or liver (right) cells transferred into either CTL (upper row) or *Pparg*^Δ/Δ^ (lower row) recipient mice. The red frames on the left and right of each plot indicate the gating and percentage of LK (Lineage-negative, Sca-1^−^CD117^+^) and LSK (Lineage-negative, Sca-1^+^CD117^+^) cells, respectively. Right panels: Relative proportions (in %) of LSK cells (top panel) and LK cells (bottom panel) over the total cell population of the BM, spleen, and liver. Dark bars represent donor control BM into CTL mice, and light bars represent donor control BM into *Pparg*^Δ/Δ^ mice. Mean ± SEM, *n* = 3 mice per genotype. All significant *p*-values are indicated above the corresponding bars.

To determine whether the EMH observed in *Pparg*^Δ/Δ^ mice was driven by a hematopoietic cell-autonomous mechanism or by a perturbation of the BM microenvironment, BM transplantation was performed. Lethally γ-irradiated CD45.1^+^ wild-type recipient mice were reconstituted with either Pparg^Δ/Δ^ or control littermate T-depleted BM cells (CD45.2^+^). After 3 months, reconstitution from the donor was verified by staining peripheral blood lymphocytes (PBLs) for the allelic markers CD45.2 and CD45.1, and the mice were sacrificed for organ analyses. When wild-type recipients were reconstituted with donor BM from *Pparg*^Δ/Δ^ or control mice, no significant difference was observed in either the number or proportions of most mature hematopoietic cell types (Supplementary Figure S5A). Furthermore, there was no evidence of EMH in these chimeras, as no increases in LSK or LK cells were observed in the spleen or the liver (Figure 3A).

We then performed the reciprocal experiment in which lethally irradiated *Pparg*^Δ/Δ^ (CD45.2^+^) recipients were reconstituted with wild-type BM from CD45.1^+^ mice (reverse chimeras). In contrast to what we observed when using WT recipients, the EMH observed in the peripheral organs of *Pparg*^Δ/Δ^ recipient mice was largely recapitulated (Figure 3B). Increased numbers and proportions of erythroblasts, granulocytes, lymphocytes, and macrophages were observed in the spleen (Supplementary Figure S5B), and liver (data not shown) in these reverse chimeras. Further evidence of EMH was provided by the increase in proportions of LSK and LK cells in the peripheral organs of *Pparg*^Δ/Δ^ recipients, but not of control recipients, reconstituted with wild-type donor BM (Figure 3B).

Taken together, these results indicate that the EMH observed in *Pparg*^Δ/Δ^ mice is non-cell autonomous. Although LT-HSCs (and other stem/progenitor subsets) appeared to home to and seed normally in the BM cavity in lethally irradiated *Pparg*^Δ/Δ^ mice, they were also able to efficiently seed peripheral organs such as the spleen and liver and to mobilize to these organs. This suggests that the resulting EMH, rather than a defect of the hematopoietic cells themselves, was driven either by systemic cues or by changes in the microenvironment or factors produced by cells within the local microenvironment.

### Respective contributions of inflammation and lipodystrophy to EMH onset

One important known systemic cause of EMH is inflammation. In a previous report, we showed that *Pparg*^Δ/Δ^ mice have altered skin hair follicles, which with aging provoke an inflammatory response in the skin (18). To evaluate the contribution of these systemic disorders in the occurrence of EMH in *Pparg*^Δ/Δ^ mice, we analyzed another mouse model that shares a similar phenotype with respect to lipodystrophy, but does not exhibit overt inflammation.

**Figure 4:**
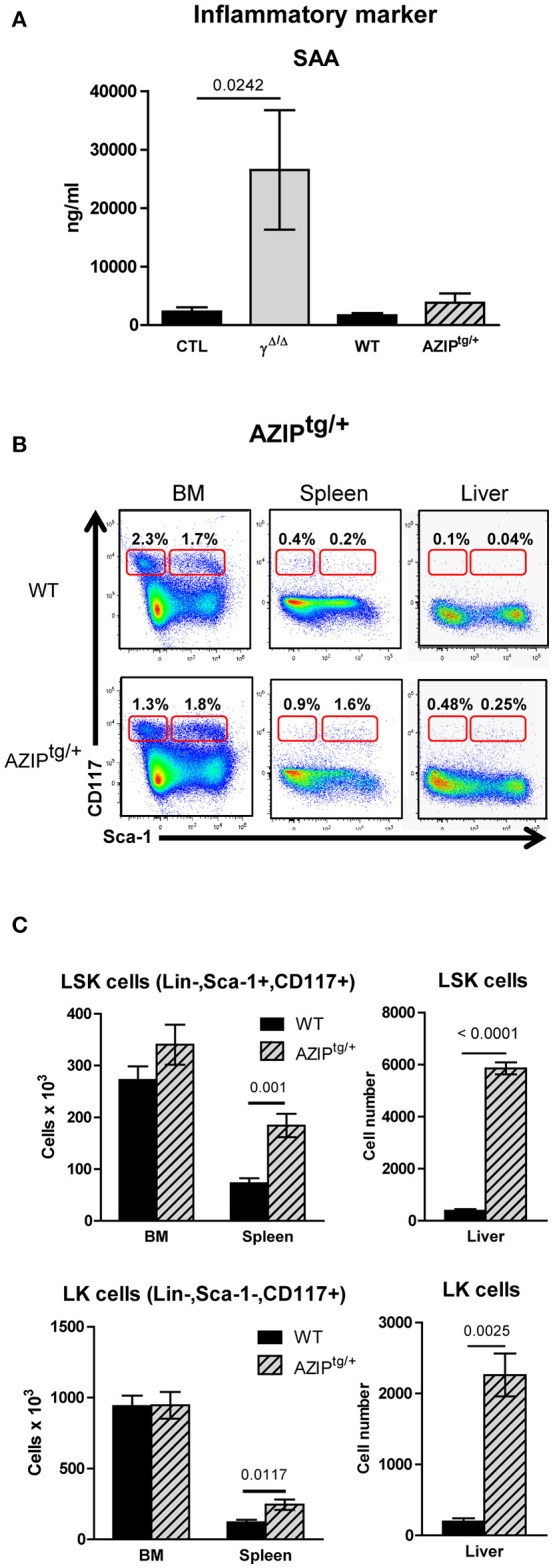
Investigation of extramedullary hematopoiesis in AZIP^tg/+^ mice. **(A)** Serum levels of the inflammatory marker Serum Amyloid A in *Pparg*^Δ/Δ^ and in *AZIP*^*tg*/+^, evaluated by ELISA. Mean ± SEM for 3 to 8 mice per genotype. **(B)** Representative FACS plots showing Sca-1 vs. CD117 staining on lineage-negative bone marrow (BM; left panels) and spleen (middle panels) and liver (right panels) cells from AZIP^tg/+^ (lower row) mice and their wild-type (WT) littermates (upper row). The red frames on the left and right of each plot indicate the gating and percentage of LK (Lineage-negative, Sca-1^−^CD117^+^) and LSK (Lineage-negative, Sca-1^+^CD117^+^) cells, respectively, expressed as a percentage of lineage-negative cells from each organ. **(C)** Histograms showing total numbers of LSK (left panel) and LK (right panel) cells in the BM, spleen and liver of WT control (dark bars) and AZIP^tg/+^ (light hatched bars) mice. Mean ± SEM, *n* = 3–6 mice per genotype. All significant *p*-values are indicated above the corresponding bars.

The AZIP^tg/+^ mouse is a hemizygous transgenic mouse strain in which a dominant negative protein (A-ZIP) expressed under the control of the adipose tissue-specific aP2 enhancer/promoter inhibits expression of members of the C/EBP and Jun families of transcription factors. AZIP^tg/+^ mice are born with no WAT and with severely decreased brown adipose tissue (19). One difference between *Pparg*^Δ/Δ^ mice and AZIP^tg/+^ mice is the presence of systemic inflammation, suggested in *Pparg*^Δ/Δ^ mice by the high levels of Serum Amyloid A (SAA) protein, which is a highly sensitive marker for inflammation particularly in the acute phase, and moderate increased levels of IL-1β. In contrast, SAA, IL-1β, and IL-6 levels were not significantly increased in AZIP^tg/+^ mice, indicating that the inflammation in these mice is very low, if not null (Figure 4A and data not shown).

We thus further analyzed the liver and the spleen of the lipodystrophic AZIP^tg/+^ mice. While these animals also displayed enlarged spleens and a significant increase in total numbers of hematopoietic mononuclear cells, these increases were predominantly due to increased numbers of myeloid and erythroid cells (Supplementary Figure S6). Importantly, the EMH observed in the absence of PPARγ was recapitulated in AZIP^tg/+^ mutants (Figure 4B), with a significant increase in the numbers of LSK cells in the spleen and the liver, and an increase of LK cells in the spleen and to a lesser extent in the liver (Figure 4C).

This observation demonstrated that even though inflammation likely contributes to the EMH observed in *Pparg*^Δ/Δ^ mice, the lipodystrophy *per se* is responsible for the EMH.

### Distinct features of the EMH in *Pparg*^Δ/Δ^ and in AZIP^tg/+^ mice

**Figure 5:**
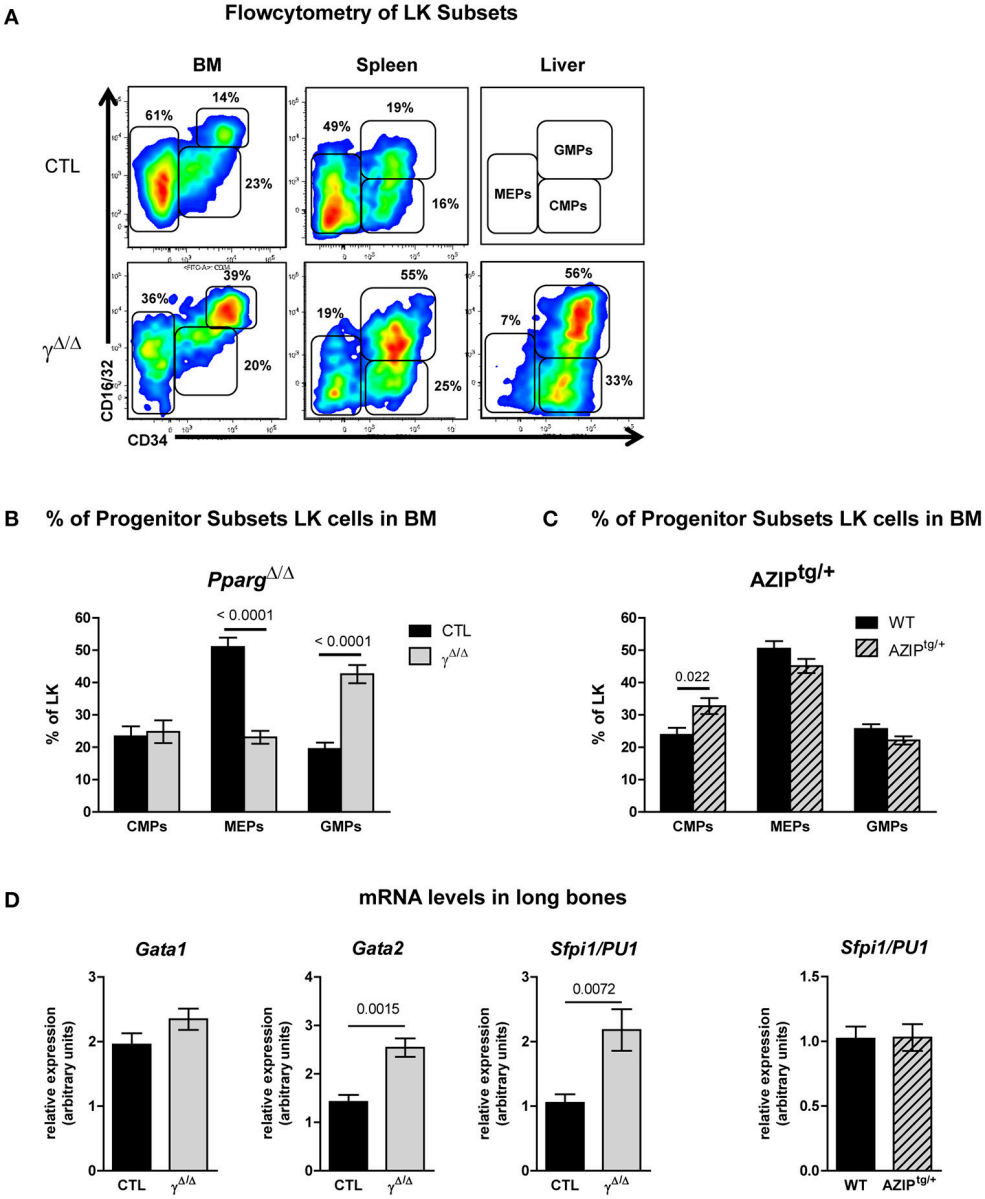
FACS analyses of progenitor cell subsets in the LK population of the bone marrow (BM), spleen, and liver of Pparg^Δ/Δ^ and AZIP^tg/+^ mice. **(A)** Representative FACS plots of CD34 vs. CD16/32 expression in the LK subset of the BM (left panels), spleen (middle panels) and liver (right panels) of control (CTL, upper row) and *Pparg*^Δ/Δ^ (lower row) mice. As there were insufficient liver cells to make a FACS plot for the CTL mice, the gating strategy for the progenitor subsets is indicated instead. The respective percentages of MEPs (CD34^−^CD16/32^−^), CMPs (CD34^low^CD16/32^−^), and GMPs (CD34^+^CD16/32^+^) are indicated in each plot. LK (Lineage-negative, Sca-1^−^,cKit-r /CD117^+^); MEP (Megakaryocyte Erythroid Progenitor); CMP (Common Myeloid Progenitor); GMP (Granulocyte Monocyte Progenitor). **(B)** Histograms showing the proportion (%) of CMPs, MEPs and GMPs in the BM LK subset of control (dark bars) and *Pparg*^Δ/Δ^ (light bars) mice. Mean ± SEM, *n* = 7–8 mice per genotype. **(C)** Histograms showing the proportion (%) of CMPs, MEPs, and GMPs in the BM LK subset of wild-type (dark bars) and AZIP^tg/+^ (light hatched bars) mice. Mean ± SEM, *n* = 7 mice per genotype. **(D)** mRNA expression levels of the transcription factors *Gata1, Gata2, Sfpi1/PU1* evaluated by qRT-PCR in total cellular extracts from the long bones of control (dark bars) vs. *Pparg*^Δ/Δ^ mice (light bars) or wild-type (dark bars) and AZIP^tg/+^ (light hatched bars) mice. Mean ± SEM, *n* = 5–7 mice per genotype. All significant *p*-values are indicated above the corresponding bars.

In the BM, inflammation provokes an increased demand on the granulocyte/macrophage lineage. This lineage is derived from one of the two cell populations that arise from the CMPs (defined as CD34^+^, CD16/32^−^): the MEPs (CD34^−^, CD16/32^+^) and the GMPs (CD34^+^, CD16/32^+^). We thus further analyzed the relative proportions of MEPs, GMPs, and CMPs found within the BM, according to these markers (see Supplementary Figure S4). In the BM, MEPs were decreased and GMPs increased, whereas CMPs were unchanged, resulting in a relatively lower proportion of MEPs and a higher proportion of granulocyte/macrophage progenitors in the BM of mice lacking PPARγ compared to wild-type controls (Figures 5A,B). These results were consistent with the gene expression profiles of their key regulators, with elevated levels of both *Sfpi/PU1* and *Gata2* in the long bones of *Pparg*^Δ/Δ^ mice compared to control mice, whereas *Gata1* remained unchanged (Figure 5D). Thus, a bias in favor of myeloid over erythroid development in the BM was observed in the absence of PPARγ.

**Figure 6:**
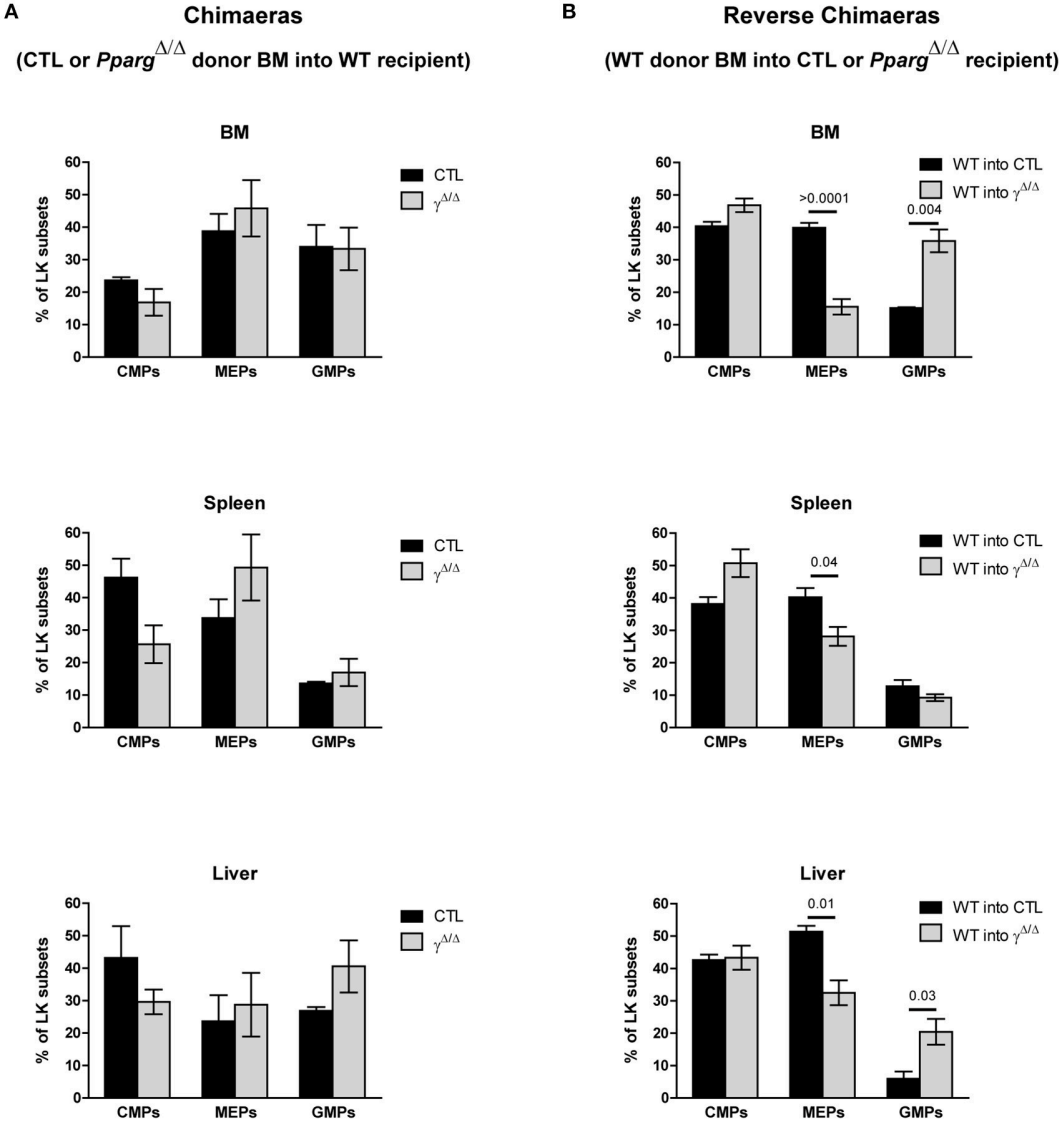
FACS analyses of progenitor cell subsets in the LK population in chimeras and AZIP^tg/+^ mice. **(A)** Chimeras using wild-type recipients and control or *Pparg*^Δ/Δ^ donor BM. Hematopoietic reconstitution was evaluated by FACS analysis 3 months after the transfer (see also Figure 3A). The histograms show the proportion (%) of CMPs (CD34^low^CD16/32^−^), MEPs (CD34^−^CD16/32^−^), and GMPs (CD34^+^CD16/32^+^) in the LK cell subset, in the BM, the spleen and the liver of WT mice reconstituted with control (dark bars) or *Pparg*^Δ/Δ^ (light bars) BM. Mean ± SEM, *n* = 3 mice per genotype. No significant *p*-value. **(B)** Reverse chimeras using wild-type (WT) control donor BM transferred into *Pparg*^Δ/Δ^ or their littermate control (CTL) recipient. The histograms show the proportion (%) of CMPs, MEPs and GMPs in the LK cell subset in the BM, the spleen and the liver of control (dark bars) or *Pparg*^Δ/Δ^ (light bars) mice reconstituted with wild-type donor BM. Mean ± SEM, *n* = 3 mice per genotype. All significant *p*-values are indicated above the corresponding bars.

Importantly, the observed shift in the ratio of GMPs to MEPs within the LK subset in *Pparg*^Δ/Δ^ mice was also recapitulated in the reverse chimeras in the BM (Figures 6A,B), when wild-type cells were used to reconstitute the BM of irradiated *Pparg*^Δ/Δ^ mice. However, in AZIP^tg/+^ mice, which harbor no inflammation, increase of the LK cell population was observed in all progenitor subsets without alteration of the MEP to GMP ratio (Figure 5C). Consistent with this observation, mRNA expression levels of the myeloid-promoting transcription factor SFPI1/PU.1 were not increased in the bones of AZIP^tg/+^ mice (Figure 5D). Altogether, these data suggest that the increased ratio of GMPs over MEPs is in part linked to the systemic inflammation, whereas the EMH is linked to the lipodystrophy context.

Another distinct feature between the phenotypes of *Pparg*^Δ/Δ^ and AZIP^tg/+^ mice is that no decrease is observed in the numbers of B lymphocytes in the BM of AZIP^tg/+^ mice (Supplementary Figures S6A,B). Further analyses in *Pparg*^Δ/Δ^ mice demonstrated more specifically that while the early immature B cell subsets (PreProB, ProPreB, PreBI and large and small PreBII) were similar in control and *Pparg*^Δ/Δ^ mice, the numbers of later-stage B cells (IgM^+^ immature and mature cells) were significantly decreased in the absence of PPARγ (data not shown). These observations, not seen in AZIP^tg/+^ mice, are therefore unlikely to be directly due to the lack of adipocytes in the BM.

### Altered HSC retention in lipodystrophic mice BM contributes to EMH in peripheral organs

The next question was therefore which common mechanism in these two models of lipodystrophy would promote EMH. One important systemic perturbation observed in these two models, which is directly due to the lack of adipose tissue, is a severe type 2 diabetes phenotype with hyperglycemia and hyperinsulinemia. To evaluate the possible impact of these metabolic disorders in the induction of EMH, we analyzed the occurrence of EMH in a well-characterized model of type 2 diabetes, the leptin-deficient *ob/ob* mice. In contrast to the *Pparg*^Δ/Δ^ and AZIP^tg/+^ mice, no increase in spleen size was observed in *ob/ob* mice (data not shown). Moreover, normal numbers of LSK and LK cells were observed in the spleen of *ob/ob* mice (Supplementary Figures S6C,D), thus ruling out metabolic perturbation as a possible cause of EMH.

As the EMH observed in *Pparg*^Δ/Δ^ mice was not due to a cell autonomous defect in hematopoietic cells, and was reproduced in another model of lipodystrophy, we hypothesized that the lack of adipocytes or factors produced by adipocytes could be responsible for this phenomenon. Indeed, one mechanism by which peripheral organs such as the liver and the spleen harbor HSC/progenitor cells is through egression of cells from the BM. This also occurs under normal homeostatic conditions, since small numbers of HSC/progenitor cells can be found, albeit at barely detectable levels, in the peripheral organs of normal mice (see for example Figures 2A–C).

**Figure 7:**
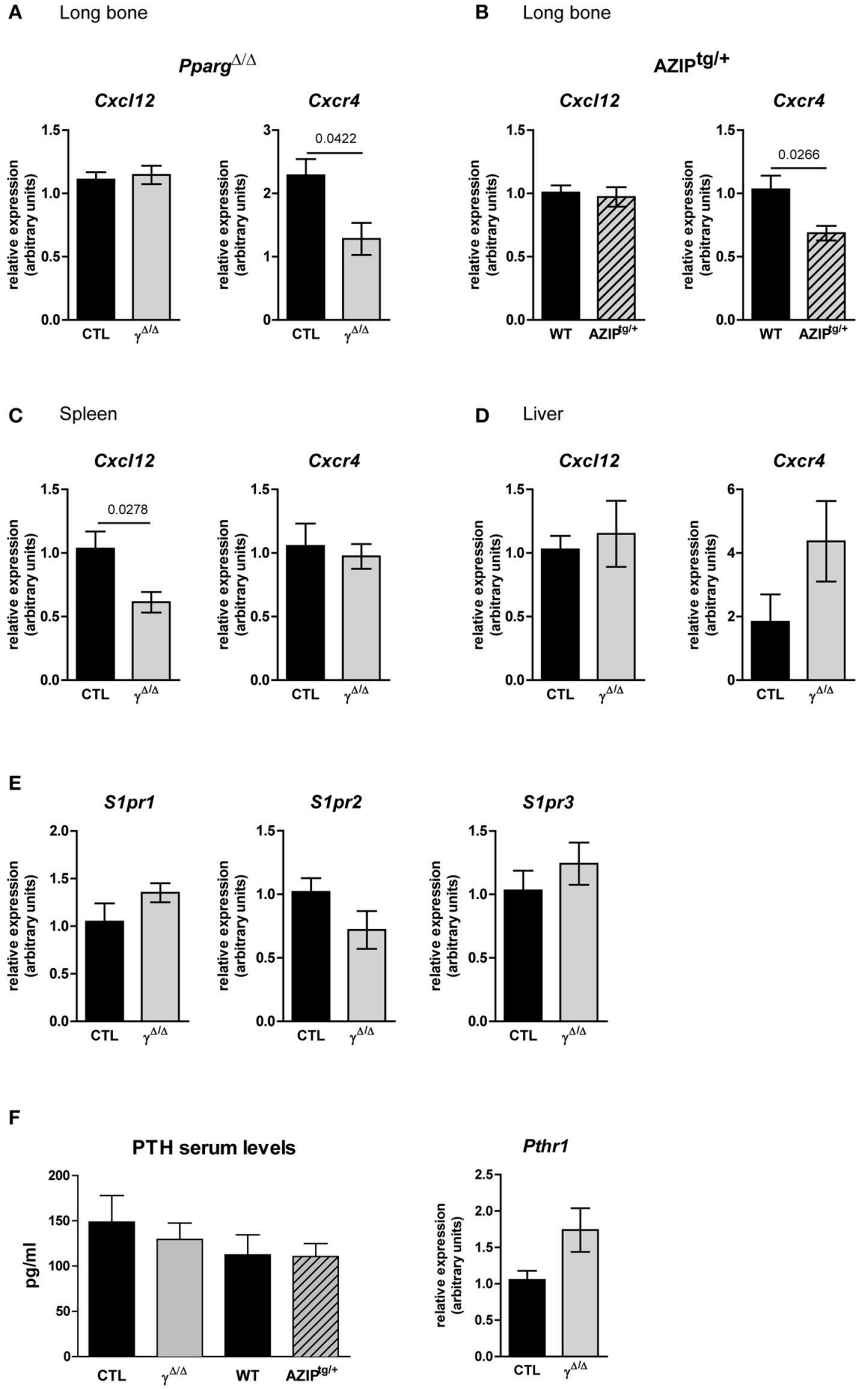
Gene expression of key factors of cell egress from the BM in Pparg^Δ/Δ^ and AZIP^tg/+^ mice. **(A,B)** *Cxcl12* and *Cxcr4* mRNA expression in the long bones of *Pparg*^Δ/Δ^ **(A)** and AZIP^tg/+^ **(B)** mice and their controls, CTL and WT, respectively, as indicated. Mean ± SEM for 3 to 6 mice per genotype. **(C,D)** *Cxcl12* and *Cxcr4* mRNA expression in the spleen **(C**; Mean ± SEM for 5 mice per genotype) and the liver **(D**; Mean ± SEM for 6 to 7 mice per genotype) of *Pparg*^Δ/Δ^ mice and their control. **(E)** Expression levels of Sphingosine Sphingosine kinase/sphingosine 1-phosphate receptors genes, *S1pr1, S1pr2, S1pr3*, in the long bone of *Pparg*^Δ/Δ^ mice and their control. **(F)** Parathyroid hormone (PTH) serum levels and gene expression of its receptor PTHR1 in long bones of *Pparg*^Δ/Δ^ mice and their control Mean ± SEM for 3 to 4 mice per genotype. All significant *p*-values are indicated above the corresponding bars.

The lack of adipocytes in the BM of *Pparg*^Δ/Δ^ and AZIP^tg/+^ mice may disrupt the local microenvironment. We thus explored whether the EMH observed in the absence of adipocytes resulted from an increase in cellular egress from the BM. As the CXCL12/CXCR4 axis is the main source of retention signals that maintain hematopoietic stem and progenitor cells (HSPCs) in the BM (26), we evaluated the expression levels of this cytokine (*Cxcl12*) and its receptor (*Cxcr4*) in mRNA isolated from the long bones. These extracts included both stromal and hematopoietic cell niche components. In the two models we studied, the levels of *Cxcl12* remained unaffected, whereas a significant reduction of *Cxcr4* was observed. Albeit it remains speculative, these results suggest that the retention of HSPCs in the BM might be impaired, contributing to the EMH in *Pparg*^Δ/Δ^ and in AZIP^tg/+^ mice. In contrast, *Cxcl12* is decreased in the spleen, whereas *Cxcr4* expression is not affected, indicating that these signals do not contribute to retaining HSCs in the spleen (Figure 7). The Sphingosine kinase/sphingosine 1-phosphate (S1P)/S1P receptor axis as well as the Parathyroid hormone (PTH) and its receptor PTHRP, two signals that play a role in this context (27, 28) are not different between the two genotypes (Figure 7), excluding their contribution to the phenotype.

Altogether, these results highlight that the occurrence of EMH in lipodystrophic mouse models results from a lack of adipocytes and is aggravated when systemic inflammation occurs.

## Discussion

In this study, we explored the role of adipocytes in BM homeostasis and regulation of hematopoiesis and showed that the total lipodystrophy in *Pparg*^Δ/Δ^ mice is accompanied by a severe EMH that impacts upon all hematopoietic lineages. After a thorough analysis of the cell lineages found in the spleen and liver, we first demonstrated that the EMH observed in *Pparg*^Δ/Δ^ mice is not a direct consequence of the lack of PPARγ in hematopoietic cells that normally express PPARγ (29, 30). Indeed, the EMH was reproduced when WT cells were used to reconstitute lethally irradiated *Pparg*^Δ/Δ^ mice. We then observed EMH in an independent model of lipodystrophy (AZIP^tg/+^). Albeit, we cannot exclude a contribution of inflammation in *Pparg*^Δ/Δ^ mice, data from both models indicate that the most likely causes of EMH are linked to the lipodystrophy with possible local alterations of the BM microenvironment. Thus, the combination of the two experimental models of lipodystrophy used, *Pparg*^Δ/Δ^ and AZIP^tg/+^, allowed us to reveal an important contribution of adiposity in setting the appropriate BM microenvironment required for normal hematopoiesis.

There are three main known causes of EMH in the clinics and in experimental settings. The first one is associated with myelofibrosis disorders, which trigger a compensation in hematopoietic organs such as the spleen and liver to maintain functional hematopoiesis (31). Primary myelofibrosis begins as a myeloproliferative disorder and leads to an altered marrow with cellular abnormalities. Along this line, EMH has also been observed in mice carrying hypomorphic *Pparg* alleles and was shown to result from structural changes in the bones and thus reduced numbers of BM cells (32). In contrast, we found here that the BM in *Pparg*^Δ/Δ^ and AZIP^tg/+^ mice exhibited close to normal cell numbers, with neither myeloproliferation nor dramatic changes in the HSC population, excluding myelofibrosis as the cause of EMH. The second main cause of EMH is hypoxia, in which the increased need of red blood cell production is the trigger. The stress response to hypoxia in the context of hemoglobinopathy (33) stimulates erythropoiesis and increases hematocrit. Again, neither increases in erythropoiesis nor increased hematocrit were observed in the absence of PPARγ or in AZIP^tg/+^ mice, making this hypothesis also unlikely.

The third main cause of EMH is the presence of severe systemic inflammation, particularly in rodents, where it is associated with a marked increase in granulopoiesis (17). Local skin inflammation is indeed observed in *Pparg*^Δ/Δ^ mice and was associated with PPARγ-dependent scarring alopecia (18). However, this feature is specific to *Pparg*^Δ/Δ^ mice and not found in AZIP^tg/+^ mice. In addition, the blood levels of the inflammatory markers are significantly increased in *Pparg*^Δ/Δ^ mice but not in AZIP^tg/+^ mice. Thus, although we cannot rule out a contribution of inflammation, particularly in the case of the *Pparg*^Δ/Δ^ mice, it does not explain the presence of EMH in AZIP^tg/+^ mice. These distinct features in terms of inflammation also provide an explanation for the biased development of CMPs toward the myeloid lineage at the expense of the erythroid and megakaryocyte lineages seen only in *Pparg*^Δ/Δ^ and not in AZIP^tg/+^ mice. The inflammation seen in the former and not in the latter likely contributes to this shift in CMP commitment. The resulting relative decrease in the number of erythroblasts in the BM of *Pparg*^Δ/Δ^ mice might thus be compensated for by active EMH.

Finally, although the three mouse models described in this study (*Pparg*^Δ/Δ^, AZIP^tg/+^, and *ob/ob*) are all affected by type 2 diabetes, the fact that *ob/ob* mice did not exhibit EMH excludes this systemic metabolic disorder as a common causative factor. Thus in this context, the most likely hypothesis is that adipocytes function to contribute to hematopoietic homeostasis in the BM.

The BM transfer experiment that we performed clearly indicates that the bone cavity micro-environment was involved in the EMH observed in our experimental models. The BM stem cell niche concept was first defined as a local microenvironment that maintains and regulates the function of stem and progenitor cells (1), and many cell types have been shown to be involved in these processes [reviewed in (2–4)]. BM adipocytes may or may not be considered as part of the niche *per se*, but they are present in large numbers and could contribute to the microenvironment in several ways. First, they play a role in bone homeostasis, which may indirectly affect the BM stem cell niche. This is due to the fact that adipocytes and osteoblasts are linked *via* their common mesenchymal progenitors, resulting in a balance between adipogenesis and osteoblastogenesis. PPARγ is central in this balance since this nuclear factor is a crucial regulator of MSC orientation toward adipocytes, as it's *ex vivo* or *in vitro* activation using synthetic agonists results in an adipogenic MSC phenotype, whereas it's pharmacological blockade or genetic deletion result in the opposite phenotype i.e., an osteoblastic phenotype (34, 35). Moreover, leptin and adiponectin, which are secreted by adipocytes, also exert a local and systemic role in bone homeostasis [reviewed in (36)]. Second, a direct function for adipocytes in supporting the proliferation of hematopoietic cells has been proposed, albeit the nature of this support function is not well described. It could combine energy sources and specific adipokines, which have been proposed to mediate various aspects of HSC maintenance, quiescence and proliferation *in vitro* (9, 37, 38). In contrast, two reports have shown an anti-correlation between the number of adipocytes present in the bone cavity and the rapidity of recovery after BM irradiation (13, 39). One possible way to reconcile these conflicting observations is that the support provided by adipocytes may differ between *in vitro* and *in vivo* conditions as proposed by Spindler et al. (40). In agreement with our results, it is tempting to speculate on a specific role for BM adipocytes in this phenotype. However, our experiments do not exclude the possibility of a systemic cue directly related to the generalized lipoatrophy.

A possible consequence of an altered BM microenvironment is a modification of the balance between active retention and mobilization of HSCs. We showed that in the absence of PPARγ, all hematopoietic lineages are increased. Thus, the most likely explanation for the observed EMH is reduced BM colonization at birth or an increase in egress of HSCs and early progenitors from the BM. This is consistent with the fact that HSCs within the BM are very mobile cells, and that a small number of HSCs are constantly released into the circulation (41). Nevertheless, the best characterized mechanism of HSC and progenitor cell egress from the BM is the one provoked by pharmacological doses of G-CSF administered to patients for stem cell mobilization prior to autologous transplantation. This involves down-regulation of CXCL12, which is expressed by perivascular MSCs and CAR cells, as well as by osteoblast lineage cells. This decreased expression prevents interaction of CXCL12 with its receptor CXCR4 expressed by HSCs and progenitors, and the retention and survival of HSCs and progenitor cells in the BM (42, 43). In the models presented herein, *Cxcl12* expression levels in the BM were not altered, but gene expression of its receptor CXCR4 was significantly impaired. Pharmacological antagonists of CXCR4 are among the main recent innovations improving HSC mobilization in patients (44). Altogether, our observations are consistent with an alteration of the BM microenvironment due to the total lack of adipocytes, with loss of retention and/or increased egress of HSCs and progenitor cells from the BM to the peripheral organs, albeit this mechanistic hypothesis remains speculative. Nevertheless, we cannot completely exclude the contribution of an increased pool of splenic HSCs during development in both models of lipodystrophic mice. This could be due to reduced BM colonization at birth, when stem cell niches are being formed, or to an increased retention in spleen and liver, which are the primary sites of hematopoiesis before birth, although the decrease in CXCL12 expression in the spleen, does not favor this hypothesis.

The main limitation of the present study originates from the fact that mice, like most rodents, are more prone to develop EMH than humans. Nonetheless, our results strongly support the fact that lack or scarcity of adipocytes is deleterious for BM hematopoiesis. A deeper analysis of the adipocytes in the BM stem cell niche itself, more particularly with respect to their paracrine activity and cell-cell contacts may help to better define the role of adipocytes in cell retention/egress from the BM and highlight the importance of some key factors of interest for clinical situations of BM transfer. Alternately, and not exclusively, the search for a systemic cue linked to the generalized lipodystrophy might provide new avenues of research in hematopoiesis.

## Author contributions

AW conceived the study, designed the experimental plan, performed all flow cytometry experiments, analyzed the data, wrote the manuscript, and acquired financial support for the project. HF designed the experimental plan, performed the experiments, analyzed the data, and contribute to the original draft preparation. MS, CW, and MK performed experiments, analyzed the data, and prepared the vizualisation. FG, NB, and J-YJ contributed to the conceptualization, analyzed the data, and reviewed/edited the manuscript. FR and SL performed histology experiments, analyzed the data, and prepared the figure for the spleen characterization. DM performed experiments, analyzed the data, contribute to the conceptualization, and reviewed/edited the manuscript. BD conceived the study, designed the experimental plan, supervised the study, analyzed the data, wrote the manuscript, and acquired financial support for the project. All authors read and edited the manuscript. AW and BD are the guarantors of this work and, as such, had full access to all the data in the study and take responsibility for the integrity of the data and the accuracy of the data analyses.

### Conflict of interest statement

The authors declare that the research was conducted in the absence of any commercial or financial relationships that could be construed as a potential conflict of interest.
