# Supplementary material for: Lack of Adipocytes Alters Hematopoiesis in Lipodystrophic Mice
Source: Front Immunol. 2018 Nov 13;9:2573. doi: 10.3389/fimmu.2018.02573 (PMC6244608; doi:10.3389/fimmu.2018.02573)
Supplement: Supplementary file 1 [file Presentation_1.PPTX]

## Slide 1
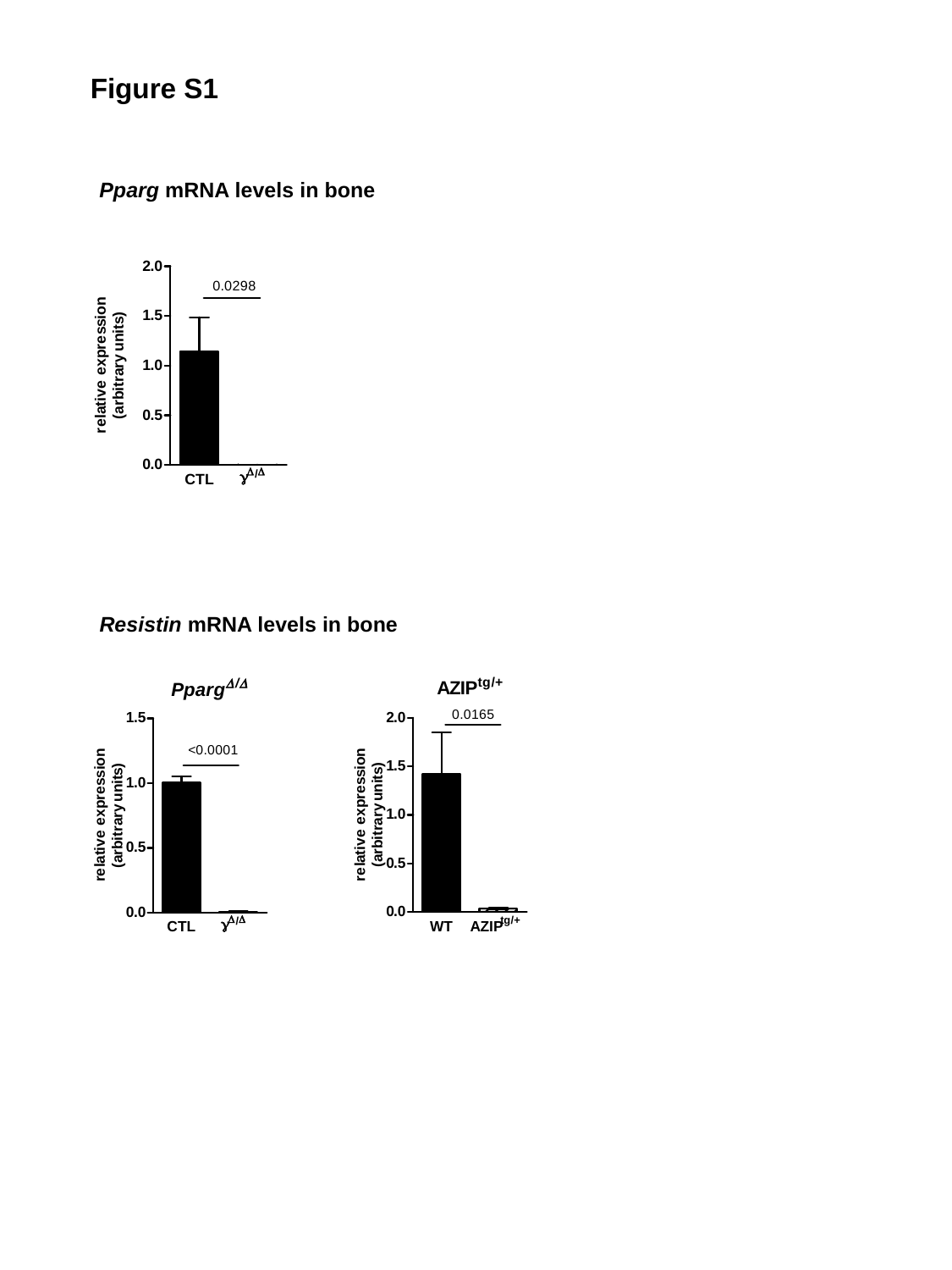

Figure S1
Pparg mRNA levels in bone
Resistin mRNA levels in bone

## Slide 2
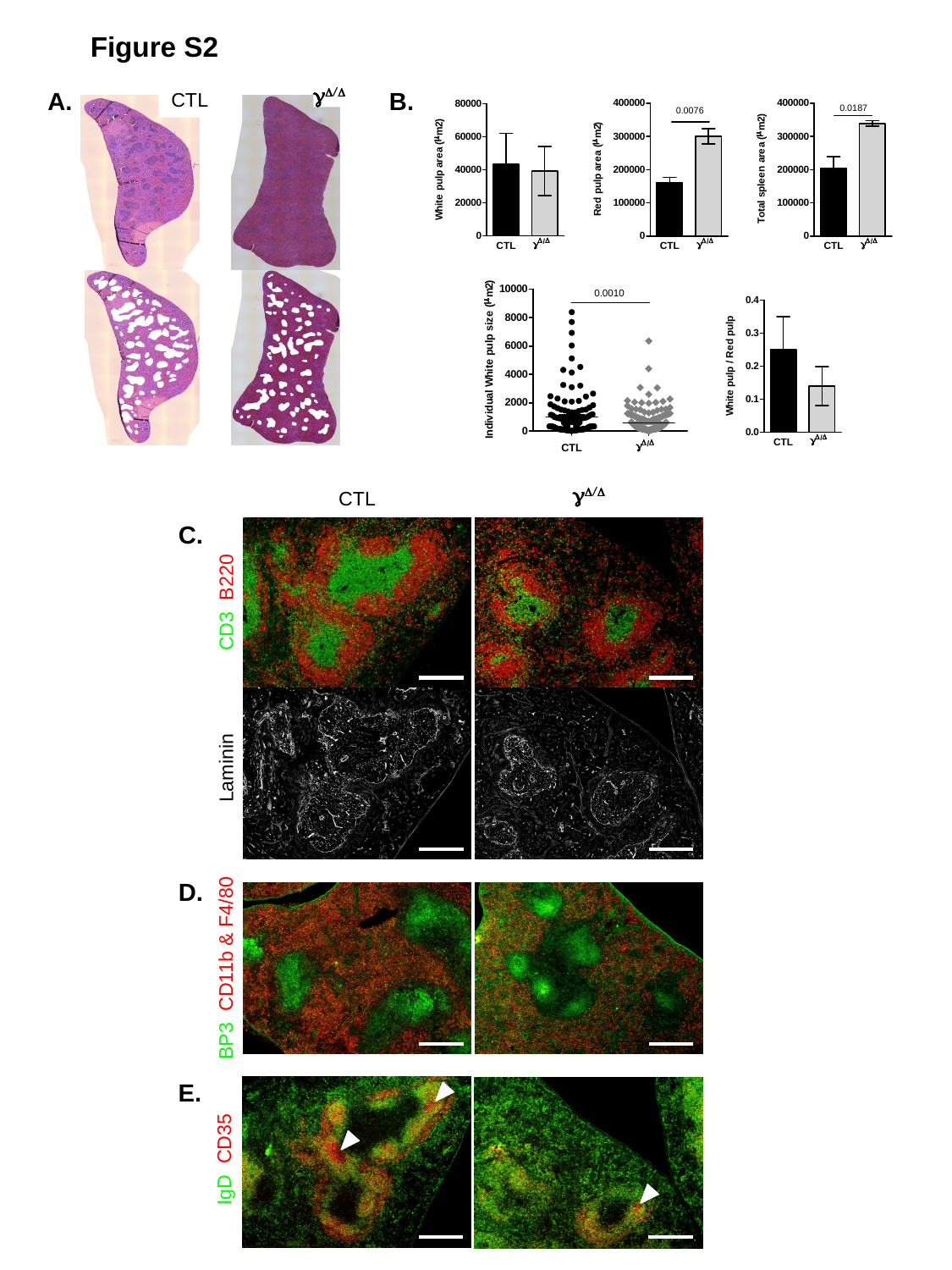

Figure S2
A.
B.
CTL
gD/D
CTL
gD/D
C.
CD3 B220
Laminin
D.
E.
IgD CD35
BP3 CD11b & F4/80

## Slide 3
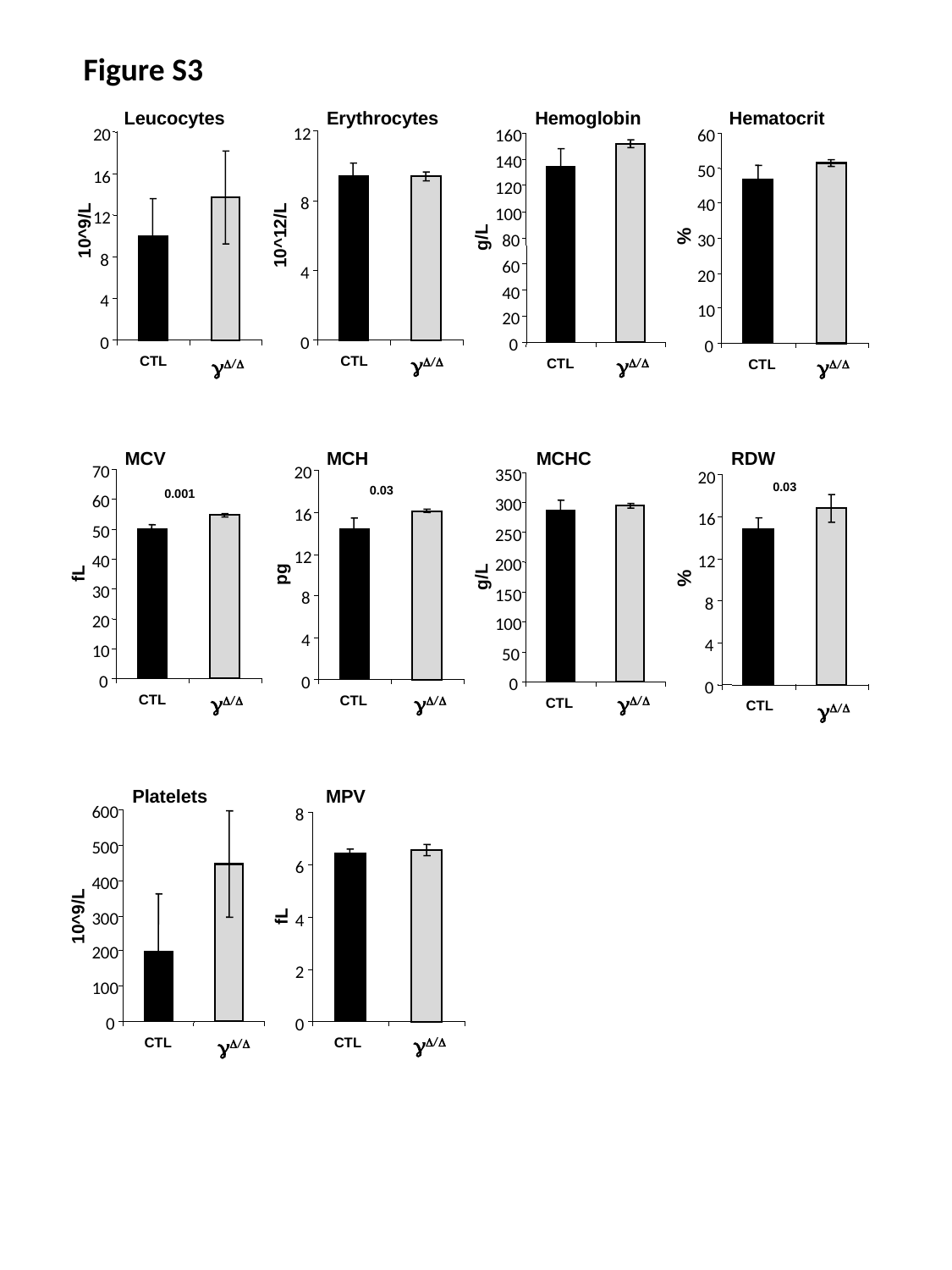

Figure S3
Leucocytes
20
16
12
10^9/L
8
4
0
CTL
gD/D
Erythrocytes
12
8
10^12/L
4
0
gD/D
CTL
Hemoglobin
160
140
120
100
g/L
80
60
40
20
0
gD/D
CTL
Hematocrit
60
50
40
%
30
20
10
0
gD/D
CTL
MCV
70
60
50
40
fL
30
20
10
0
gD/D
CTL
MCH
20
0.03
16
12
pg
8
4
0
gD/D
CTL
MCHC
350
300
250
200
g/L
150
100
50
0
gD/D
CTL
RDW
20
0.03
16
12
%
8
4
0
CTL
gD/D
0.001
Platelets
600
500
400
10^9/L
300
200
100
0
gD/D
CTL
MPV
8
6
fL
4
2
0
gD/D
CTL

## Slide 4
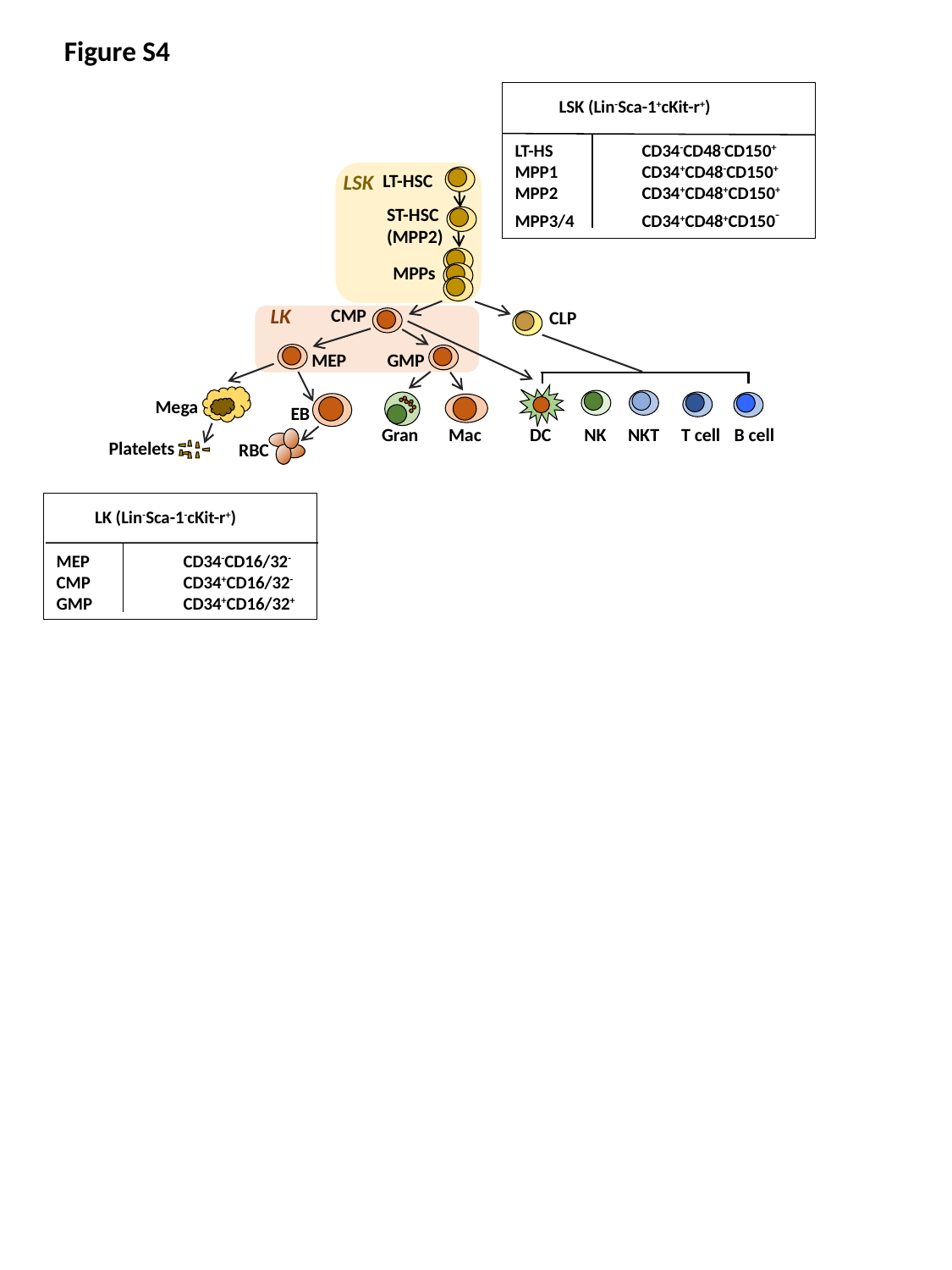

Figure S4
 LSK (Lin-Sca-1+cKit-r+)
LT-HS	CD34-CD48-CD150+
MPP1	CD34+CD48-CD150+
MPP2	CD34+CD48+CD150+
MPP3/4	CD34+CD48+CD150-
LSK
LT-HSC
ST-HSC
(MPP2)
MPPs
CMP
CLP
MEP
GMP
Mega
EB
Gran
Mac
DC
NK
NKT
T cell
B cell
Platelets
RBC
LK
 LK (Lin-Sca-1-cKit-r+)
MEP	CD34-CD16/32-
CMP	CD34+CD16/32-
GMP	CD34+CD16/32+

## Slide 5
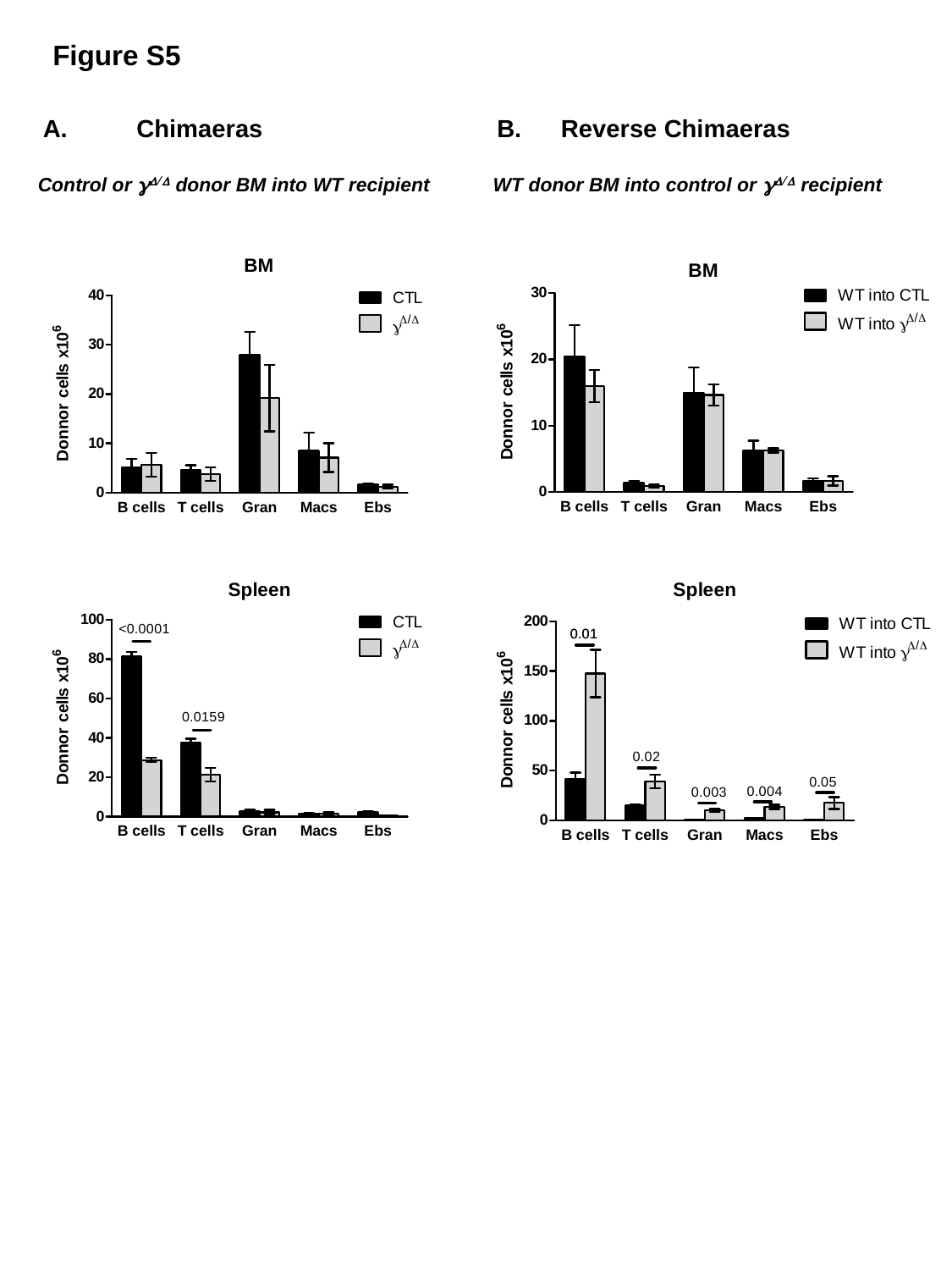

Figure S5
B.
A.
Chimaeras
Reverse Chimaeras
Control or gD/D donor BM into WT recipient
WT donor BM into control or gD/D recipient

## Slide 6
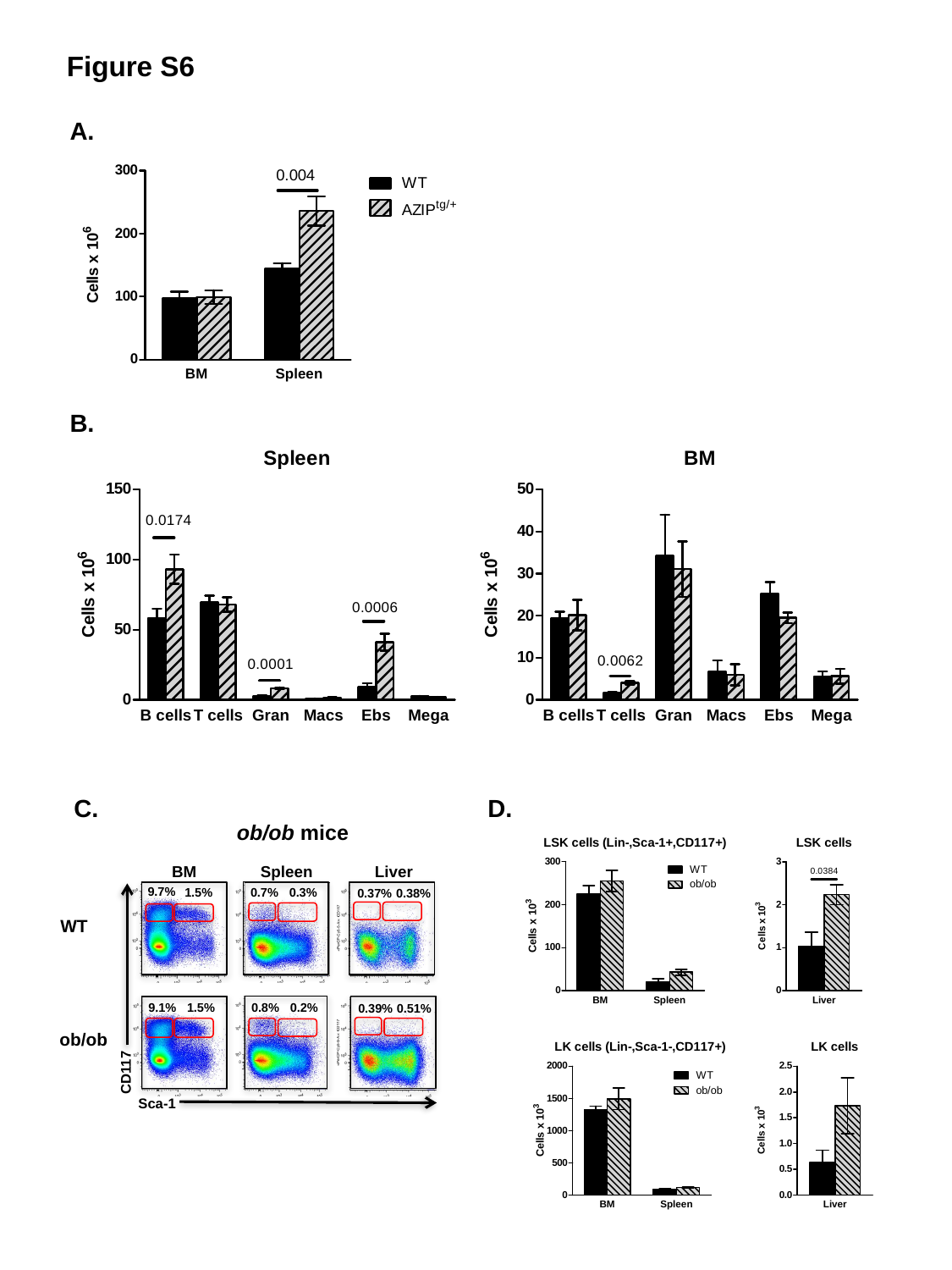

Figure S6
A.
B.
D.
C.
ob/ob mice
BM
Spleen
Liver
9.7%
1.5%
0.7%
0.3%
0.37%
0.38%
WT
9.1%
1.5%
0.8%
0.2%
0.39%
0.51%
ob/ob
CD117
Sca-1
