# Supplementary material for: Lack of Adipocytes Alters Hematopoiesis in Lipodystrophic Mice
Source: Front Immunol. 2018 Nov 13;9:2573. doi: 10.3389/fimmu.2018.02573 (PMC6244608; doi:10.3389/fimmu.2018.02573)
Supplement: Supplementary file 2 [file Data_Sheet_1.pdf]

Supplementary Figure Legends

Figure S1. Bone marrow adiposity in Pparg$^{Δ/Δ}$ and A-ZIP$^{tg/+}$ mice and their littermate controls.

Relative mRNA levels of the adipose tissue-specific adipokine *Resistin* measured in the bones of Control (CTL, dark bars) and *Pparg*$^{Δ/Δ}$ (light bars) mice, left panel; and in Wild-type (WT, dark bars) and AZIP$^{tg/+}$ (light bars) mice, right panel. Mean ± SEM for 3 to 6 mice per genotype.

Figure S2: Characterization of the spleen organization in Pparg$^{Δ/Δ}$ mice.

(A) Images of representative spleen sections stained by Haematoxilin/Eosin (top panels). White pulp (WP) areas are shown in the bottom panels as white surfaces to illustrate the method by which the surfaces have been measured. **(B)** Total spleen area, and WP areas measured from images as shown in A. The red pulp (RP) area is calculated (total section area-minus the white pulp areas). Shown below is a scatter plot containing all individual WP areas (left bottom panel) demonstrating that the mean size of the individual WP areas is smaller in the *Pparg*$^{Δ/Δ}$ ($γ^{Δ/Δ}$) mice compared to control mice (CTL). The difference in the ratio WP/RP in *Pparg*$^{Δ/Δ}$ ($γ^{Δ/Δ}$) mice compared to control mice (CTL) is not statistically significant (right bottom panel). **(C-E)** Representative immunostainings of spleen sections showing a fairly conserved structure of this lymphoid organ. **(C)** Upper images show the distribution of B220+ B cells and CD3+ T cells with most cells localizing correctly within the B and T zone, respectively, of the WP in both strains. Lower images show the same sections stained for the extracellular matrix component laminin (in white) that demarcates the WP versus RP. **(D)** Distribution of CD11b+ and/or F4/80+ myeloid cells which localize to the RP. The WP is visualized by the staining for BP3+ fibroblasts. **(E)** IgD staining for naïve B cells showing the clustering of these cells in follicles. CD35 stains most follicular fibroblasts and many B cells. Spontaneous germinal centers are represented by IgD-CD35+ areas (arrow) that are surrounded by an IgD+ follicular mantle. Scale bar = 200µm. N=3 for each genotype.

Figure S3. Blood cell counts in Pparg$^{Δ/Δ}$ mice and their littermate controls.

Blood cell counts were obtained in 12 weeks-old mice $Pparg^{\Delta/\Delta}$ mice and their littermate controls (CTL). MCV (Mean Corpuscular Volume); MCH (Mean Corpuscular Haemoglobin); MCHC (Mean Corpuscular Haemoglobin Concentration); RDW (Red Cell Distribution Width); MPV (Mean Platelet Volume). The results are mean ± SD for 19 control and 3 $Pparg^{\Delta/\Delta}$ mice.

Figure S4. Schematic diagram of haematopoiesis.

Schematic showing the classical developmental pathway of mature haematopoietic lineages from LT-HSC in the BM. LSK (Lineage-negative, Sca-1^{+},cKit-r/CD117^{+}); LK (Lineage-negative, Sca-1^{-},cKit-r/CD117^{+}); LT-HSC (Long-Term Haematopoietic Stem Cell); ST-HSC (Short-Term Haematopoietic Stem Cell); MPP (Multi-Potent Progenitor); CMP (Common Myeloid Progenitor); MEP (Megakaryocyte Erythroid Progenitor); GMP (Granulocyte Monocyte Progenitor); Mega (Megakaryocyte; CD41^{+}); RBC (Red Blood Cell; Ter119^{+}CD71^{-}); Gran (Granulocyte; Gr1^{+}CD11b^{+}); Mac (Macrophage; Gr1^{-}CD11b^{+}); CLP (Common Lymphoid Progenitor; Lin-CD135^{+}CD127^{+}); DC (Dendritic Cell; CD11c^{+}); NK (Natural Killer; CD3^{-}CD161^{+}); NKT (NK T cell; CD3^{+}CD161^{+}). The associated tables described the markers used for identifying the various HSC, MPP and progenitor subsets depicted.

Figure S5. Reconstitution of mature haematopoietic subsets by donor bone marrow (BM) in the BM and spleen of chimaeras.

(A) Chimeras using wild-type recipient: CD45.2^{+} control (CTL) or $Pparg^{\Delta/\Delta}$ donor BM transferred into lethally irradiated WT (CD45.1^{+}) hosts. Total numbers of CD45.2^{+} donor-derived mature haematopoietic subsets in the BM (2 femurs and 2 tibias per mouse (top panel)) and spleen (bottom panel) of CTL (black bars) and $Pparg^{\Delta/\Delta}$ (grey bars) mice. Mean ± SEM for 3-4 mice per genotype. (**B)** Reverse chimeras: CD45.1^{+} WT BM transferred into lethally irradiated CD45.2^{+} CTL or $Pparg^{\Delta/\Delta}$ hosts. Total number of CD45.1^{+} donor-derived mature haematopoietic subsets in the BM (2 femurs and 2 tibias per mouse (top panel)) and spleen (bottom panel) of control (black bars) and $Pparg^{\Delta/\Delta}$ (grey bars) mice. Mean ± SEM for 3-4 mice per genotype. B-cells (CD19^{+}); T-cells (CD3^{+}); Gran (granulocyte, Gr1^{+}CD11b^{+}); Macs (macrophages, Gr1^{-}CD11b^{+}); Ebs (erythroblasts, CD71^{+}Ter119^{+}).
All significant p values are indicated above the corresponding bars.

Figure S6. Lipodystrophic A-ZIP$^{tg/+}$ mice have normal bone marrow (BM) but enlarged haematopoietic subsets in peripheral organs.

(A) Total cell numbers in the BM (2 femurs and 2 tibias per mouse) and spleen of control WT (black bars) and A-ZIP$^{tg/+}$ (grey bars) mice. Mean ± SEM for 6 mice per genotype. (**B)** Total numbers of mature haematopoietic subsets in the spleen (left panel) and BM (right panel) of WT (black bars) and A-ZIP$^{tg/+}$ (grey bars) mice. Mean ± SEM for 6 mice per genotype. B-cells (CD19^{+}); T-cells (CD3^{+}); Gran (granulocyte, Gr1^{+}CD11b^{+}); Macs (macrophages, Gr1^{-}CD11b^{+}); Ebs (erythroblasts, CD71^{+}Ter119^{+}). **(C)** Representative FACS plots showing Sca-1 versus CD117 staining on lineage-negative BM (left panels), spleen (middle panels) and liver (right panels) cells from WT (upper panels) or *ob/ob* mice (lower panels). The red frames on the left and right of each plot indicate the numbers of LK (Lineage-negative, Sca-1^{-}CD117^{+}) and LSK (Lineage-negative, Sca-1^{+}CD117^{+}) cells, respectively, as a percentage of lineage-negative cells from each organ. **(D)** Histograms showing the total numbers of LSK (top panel) and LK (bottom panel) cells in the BM, spleen and liver of WT (upper panels) or *ob/ob* mice. Data are mean ± SEM from 3 mice per genotype.

All significant p values are indicated above the corresponding bars.

Supplementary Tables

Sup. Table S1. Monoclonal antibody conjugates and reagents as well as polyclonal secondary antibodies.

Antigen	Clone	Conjugates	Supplier
B220	RA3-6B2	PE-Texas Red	Pharmingen (1)
B220	RA3-6B2	APC-efluoro 780	eBioscience (1)
B220	RA3-6B2	-	In house (2)
CD3	145-2C11	-	In house, eBioscience (2)
CD4	RM4-5	PE-Cy7<br>APC-efluoro 780	eBioscience (1)
CD8a	53.6.7	Alexa 647	LICR (1)
CD8a	53.6.7	APC-efluoro 780	eBioscience (1)
CD11b	M1/70	Alexa 700<br>APC-efluoro 780	eBioscience (1)<br>(2)
CD16/32	93	PE	eBioscience (1)
CD19	1D3	PE Cy7	eBioscience (1)
CD21	7GB	FITC	BD Pharmingen (1)
CD23	B3B4	PE	BD Pharmingen (1)
CD25	PC.61	Pacific Blue	eBioscience (1)
CD27	A7R34	FITC	eBioscience (1)
CD34	RAM34	FITC	eBioscience (1)
CD35	8C12	-	In house, Biolegend (2)
CD41	MWReg30	PE	eBioscience (1)
CD43	R2/60	FITC	eBioscience (1)
CD44	IM781	APC-efluoro 780	eBioscience (1)
CD45.1	A20	PE Cy5	eBioscience (1)
CD45.2	104	efluoro 450	eBioscience (1)
CD48	HM48.1	Pacific Blue	Biolegend (1)
CD71	R17 217.1.4	FITC	eBioscience (1)
CD93	AA4.1	APC	eBioscience (1)
CD117	2B8	PerCp Cy5.5	eBioscience (1)
CD127	LG.7F9	Biotin	eBioscience (1)
CD135	A2F10	Alexa 647	eBioscience (1)
CD150	TC15-12F12.2	PE Cy5	Biolegend (1)
CD161	PK136	FITC	eBioscience (1)
BP1	6C3	PE	eBioscience (1)
BP3	BP-3.4	biotin	In house (2)
F4/80	F4/80	-	In house (2)
Gr1	RB6-8C5	Pacific Orange	Life Technologies (1)
Gr1	RB6-8C5	APC-efluoro 780	eBioscience (1)
IgD	polyclonal	-	GeneTex (2)
IgM	eB121-15F9	PerCp Cy5.5	eBioscience (1)
Laminin	polyclonal	-	Sigma (2)
Sca-1	D7	PE Cy7	eBioscience (1)
TCRβ	H57	PE	eBioscience (1)
TCRγδ	GL3	PE-Cy5	eBioscience (1)
Ter-119	TER-119	APC-efluoro 780	eBioscience (1)
Streptavidin	-	Alexa 488	Molecular Probes (2)
DAPI			Invitrogen (1)

**Secondary antibodies to:**	**source**	**conjugates**	**Supplier**
Sheep IgG	Donkey	Alexa 488	Jackson Immunoresearch (2)
Rat IgG	Donkey	Alexa 647, Cy3	Jackson Immunoresearch (2)
Rabbit IgG	Donkey	Alexa 647	Invitrogen (2)
Armenian hamster	Goat	biotin	Jackson Immunoresearch (2)

Monoclonal antibody conjugates used for flow cytometry (1) and for Immunohistochemistry (2). Antigen designation, clone name, conjugate used and supplier are listed for all monoclonal antibodies used.

Sup. Table S2. Primers used for q-RT-PCR analyses.

Gene	Fwd	Rev
Gata1	CAGAACCGGCCTCTCATCC	TAGTGCATTGGGTGCCTGC
Gata2	TAGTGCATTGGGTGCCTGC	AGGTGGTGGTTGTCGTCTGA
PU1	AGAAGCTGATGGCTTGGAGC	GCGAATCTTTTTCTTGCTGCC
Cxcl12	CGC TCT GCA TCA GTG ACG	TGAAGGGCACAGTTTGGAG
Cxcr4	ATGGAACCGATCAGTGTGAG	GATGAAGTAGATGGTGGGCAG
Resistin	GGCTTAAATTGCTGGACAGTC	TCTATCCTTGCACACTGGC
S1pr1	AGCCCTCTCGGACCTATTAG	AGCCCTCTCGGACCTATTAG
S1pr2	TGGCTGATATCGCTGATT CTG	GCCAGTAAGATGACGGAGAAG
S1pr3	GGAGGGCAGTATGTTCGTAG	AGCACATCCCAATCAGAAGG
Pth1r	CCCCGAGTCTAAAGAGAACAAG	GTAATCGGGACAAGGTACTGC
Actb	CTAAGGCCAACCGTGAAAAGAT	CACAGCCTGGATGGCTACGT
Pparg del	AAGAGCTGACCCAATGGTTG	GCATCCTTCACAAGCATGAA

Sup. Table S3. Colony Forming Cell (CFC) assay shows EMH occurring in the spleen.

		CFU-GEMM	CFU-GM	CFU-M	CFU-G	BFU-E
**BM**	**mean**	**0**	**9**	**11**	**5**	**1**
**(CTL)**	***SEM***	***0***	***0.88***	***1.45***	***1.20***	***0.58***
**BM**	**mean**	**0**	**14**	**11**	**11**	**4**
**($\gamma^{\Delta/\Delta}$)**	***SEM***	***0***	***0.88***	***2.60***	***1.33***	***0.88***
**spleen**	**mean**	**1**	**3**	**6**	**3**	**5**
**(CTL)**	***SEM***	***0.33***	***0.88***	***0.88***	***1.00***	***1.45***
**spleen**	**mean**	**5**	**28**	**36**	**39**	**16**
**($\gamma^{\Delta/\Delta}$)**	***SEM***	***1.20***	***4.91***	***5.46***	***6.33***	***3.00***

CFC assays were performed in triplicate on BM (top 2 lanes) and spleen (bottom 2 lanes) progenitors. Splenocytes ($3x10^5$) and BM cells ($2x10^4$) cells per dish. Colonies were counted 10-12 days after seeding. Numbers correspond to the number of colonies per 35mm dishes. CFU (colony forming unit); CFU-G (CFU-granulocyte); CFU-M (CFU-macrophage); CFU-GM (CFU-granulocyte/macrophage); CFU-GMME (CFU-granulocyte/macrophage/megakaryocyte/erythroid); Burst forming unit (BFU)-erythroid.
